# Don’t Let It Get Under Your Skin! – Vaccination Protects the Skin Barrier of Common Carp From Disruption Caused by Cyprinid Herpesvirus 3

**DOI:** 10.3389/fimmu.2022.787021

**Published:** 2022-01-31

**Authors:** Mikolaj Adamek, Marek Matras, Alexander Rebl, Magdalena Stachnik, Alberto Falco, Julia Bauer, Anne-Carina Miebach, Felix Teitge, Verena Jung-Schroers, Muhammad Abdullah, Torben Krebs, Lars Schröder, Walter Fuchs, Michal Reichert, Dieter Steinhagen

**Affiliations:** ^1^ Fish Disease Research Unit, Institute for Parasitology, University of Veterinary Medicine Hannover, Hannover, Germany; ^2^ Laboratory of Fish Diseases, National Veterinary Research Institute, Pulawy, Poland; ^3^ Fish Genetics Unit, Research Institute for Farm Animal Biology (FBN), Institute of Genome Biology, Dummerstorf, Germany; ^4^ Institute of Research, Development, and Innovation in Healthcare Biotechnology in Elche (IDiBE), Miguel Hernández University (UMH), Elche, Spain; ^5^ Institute of Molecular Virology and Cell Biology, Friedrich-Loeffler-Institut, Greifswald-Insel Riems, Germany

**Keywords:** cyprinid herpesvirus 3, CyHV-3, koi herpesvirus, KHV, live attenuated virus, vaccination, secondary bacterial infections, Sbi

## Abstract

Vaccination is the best form of protecting fish against viral diseases when the pathogen cannot be contained by biosecurity measures. Vaccines based on live attenuated viruses seem to be most effective for vaccination against challenging pathogens like *Cyprinid herpesvirus 3*. However, there are still knowledge gaps how these vaccines effectively protect fish from the deadly disease caused by the epitheliotropic CyHV-3, and which aspects of non-direct protection of skin or gill integrity and function are important in the aquatic environment. To elucidate some elements of protection, common carp were vaccinated against CyHV-3 using a double deletion vaccine virus KHV-T ΔDUT/TK in the absence or presence of a mix of common carp beta-defensins 1, 2 and 3 as adjuvants. Vaccination induced marginal clinical signs, low virus load and a minor upregulation of *cd4*, *cd8* and *igm* gene expression in vaccinated fish, while neutralisation activity of blood serum rose from 14 days post vaccination (dpv). A challenge infection with CyHV-3 induced a severe disease with 80-100% mortality in non-vaccinated carp, while in vaccinated carp, no mortality was recorded and the virus load was >1,000-fold lower in the skin, gill and kidney. Histological analysis showed strongest pathological changes in the skin, with a complete destruction of the epidermis in non-vaccinated carp. In the skin of non-vaccinated fish, T and B cell responses were severely downregulated, inflammation and stress responses were increased upon challenge, whereas vaccinated fish had boosted neutrophil, T and B cell responses. A disruption of skin barrier elements (tight and adherence junction, desmosomes, mucins) led to an uncontrolled increase in skin bacteria load which most likely exacerbated the inflammation and the pathology. Using a live attenuated virus vaccine, we were able to show that increased neutrophil, T and B cell responses provide protection from CyHV-3 infection and lead to preservation of skin integrity, which supports successful protection against additional pathogens in the aquatic environment which foster disease development in non-vaccinated carp.

## Introduction

Herpesviruses are master manipulators of immune responses, which they can circumvent or even employ for their own use, to be replicated more effectively in infected cells (1). Some researchers even consider herpesviruses as symbiotic organisms which modulate host immunity in a beneficial way, protecting them from other pathogens (2). However, there is a group of herpesviruses belonging to the family *Alloherpesviridae*, which infect fish and seem to be less pleasant for their hosts, since they have an opposite effect of fostering coinfections. Some Alloherpesviruses, like *Cyprinid herpesvirus 2* and *3* (CyHV-2 and CyHV-3), cause extremely serious diseases, which lead to mass mortality in *Carassius* sp. or *Cyprinus* sp. populations (3). They still possess the ability to manipulate the immunity of their hosts, but also cause vast pathological changes in the body of the host, which lead to mortality.

CyHV-3, also known as koi herpesvirus (KHV), is the causative agent of lethal koi herpesvirus disease (KHVD) in common carp. The virus possesses an epitheliotropic nature, at least at the start of the infection, and infects the skin, gills and the intestinal tract of carp (4–7). The infection disrupts the mucosal barrier of the skin, leading to the occurrence of so-called “sandpaper skin” lacking mucus (8). Molecular studies have shown that the virus infection leads to a downregulation of mucins, antimicrobial peptides like beta-defensins and a loosening of cell-to-cell contacts (9). The infection spreads rapidly, also to internal tissues, and most likely leads to multi-organ failure. Combined pathological changes in gills and the kidney lead to a disruption of the osmotic balance in infected carp, with a severe drop in sodium levels to a concentration below 90 mmol/L, most likely resulting in death (10). Failing mucosal barriers of the skin and gills allow secondary pathogens to thrive, and thus increase the probability of secondary bacterial infections (SBI) occurring (9, 10). Therefore, it could be hypothesised that the degree of the virulence of epitheliotropic cyprinid herpesviruses is related to the specific character of the aquatic environment, where the mucosal integrity is essential for osmoregulation and protection of fish from potentially hostile bacteria present in the surrounding water (11, 12).

Vaccination is the best form of preventing outbreaks of diseases caused by pathogens not containable by biosecurity. Creating a highly efficient vaccine to fish herpesviruses belonging to the *Alloherpesviridae* family was elusive, because these viruses possess large DNA genomes, which encode at least several immunogenic proteins and cause both very severe clinical signs in their host and latency in survivors. Therefore, current biotechnological advances in creating single target vaccines, e.g. DNA-based vaccines seem to be less effective (13). A breakthrough came with the generation of live attenuated vaccines against CyHV-3. Already first studies on creating a recombinant by inserting the firefly luciferase (LUC) cassette into the TK gene of CyHV-3 (ORF 55) gave a surprisingly attenuated virus (5) and raised hopes for the development of a viable vaccine. Further studies resulted in the double gene deletion recombinant CyHV-3 FL Δ56-57, which became a very effective vaccine, protecting carp from KHVD (14). Additional vaccine candidates were developed by other laboratories, including the double deletion virus for ORF55 (thymidine kinase, TK) and ORF123 (deoxyuridine-triphosphatase, DUT). Based on a Taiwanese isolate, the KHV-T ΔDUT/TK vaccine candidate virus induced a strong protection of carp against KHVD (15).

These live attenuated vaccines were highly effective and provided up to 100% relative rate of protection from mortality associated with CyHV-3 infection (13–15). For both the double ORF56-57 deletion (14) and the KHV-T ΔDUT/TK deletion viruses (15), the efficacy and safety aspects of the attenuated recombinant vaccine were documented and the induction of a virus-specific antibody response in vaccinated carp was confirmed for the KHV-T ΔDUT/TK deletion virus (15). Furthermore, the vaccination of carp with CyHV-3 FL Δ56-57 restricted the replication of a virulent wild type virus to the skin of the challenged carp, which indicates a protective mucosal immune response at the portal of entry of the virus (14). However, the mechanistic basis of protection still remains unclear.

We hypothesised that the most profound protective effect of vaccination develops in mucosal tissues like skin and gills, protecting their integrity and function. Therefore, we analysed mucosa responses in carp vaccinated with KHV-T ΔDUT/TK (15) and challenged by infection with a hyper-virulent wild type virus associated with disruption of the skin barrier (9). The influence of CyHV-3 vaccination on immune responses against CyHV-3, skin and gill integrity as well as the development of additional secondary bacterial infections was studied.

## Material and Methods

### Naïve Common Carp

Naïve individuals from the Prerov scaly carp (PS) strain were raised as described earlier (16). For the vaccination and challenge experiments, the carp were transported to the National Veterinary Research Institute in Pulawy, Poland at the age of three years with a mean weight of 125.6 ± 54.6 g. There, the carp were acclimated for two weeks at 23°C in a flow-through keeping system prior to the vaccination experiment. After transport, tissue pools containing gills and kidneys were collected from n=5 carp and confirmed to be free of DNA or RNA-specific for CyHV-3, spring viremia of carp virus (SVCV), carp edema virus (CEV) and the common carp paramyxovirus (CCPV) as described earlier (16). All animal experiments were performed in accordance with national and international regulations for experimentation with animals and under approval 32/2020 of the Local Ethical Committee in Lublin, Poland.

### Application of Adjuvants

The mix of synthetically generated mature peptides for common carp defensin beta 1, beta 2 and beta 3 proteins were used as a bath adjuvant two days before the vaccination. After synthesis, the peptides were refolded as described by Wu et al. (17). The final solution containing 1 μg mL^-1^ was applied in the bath treatment for 1h. As a control, 1 μg mL^-1^ lipid-free bovine serum albumin (BSA) was used for mock treatment. BSA was selected as an inert protein source, which was shown to be safe in baths of fish in 20,000-fold higher concentrations than used in current work (18).

### Vaccination

**Figure 1:**
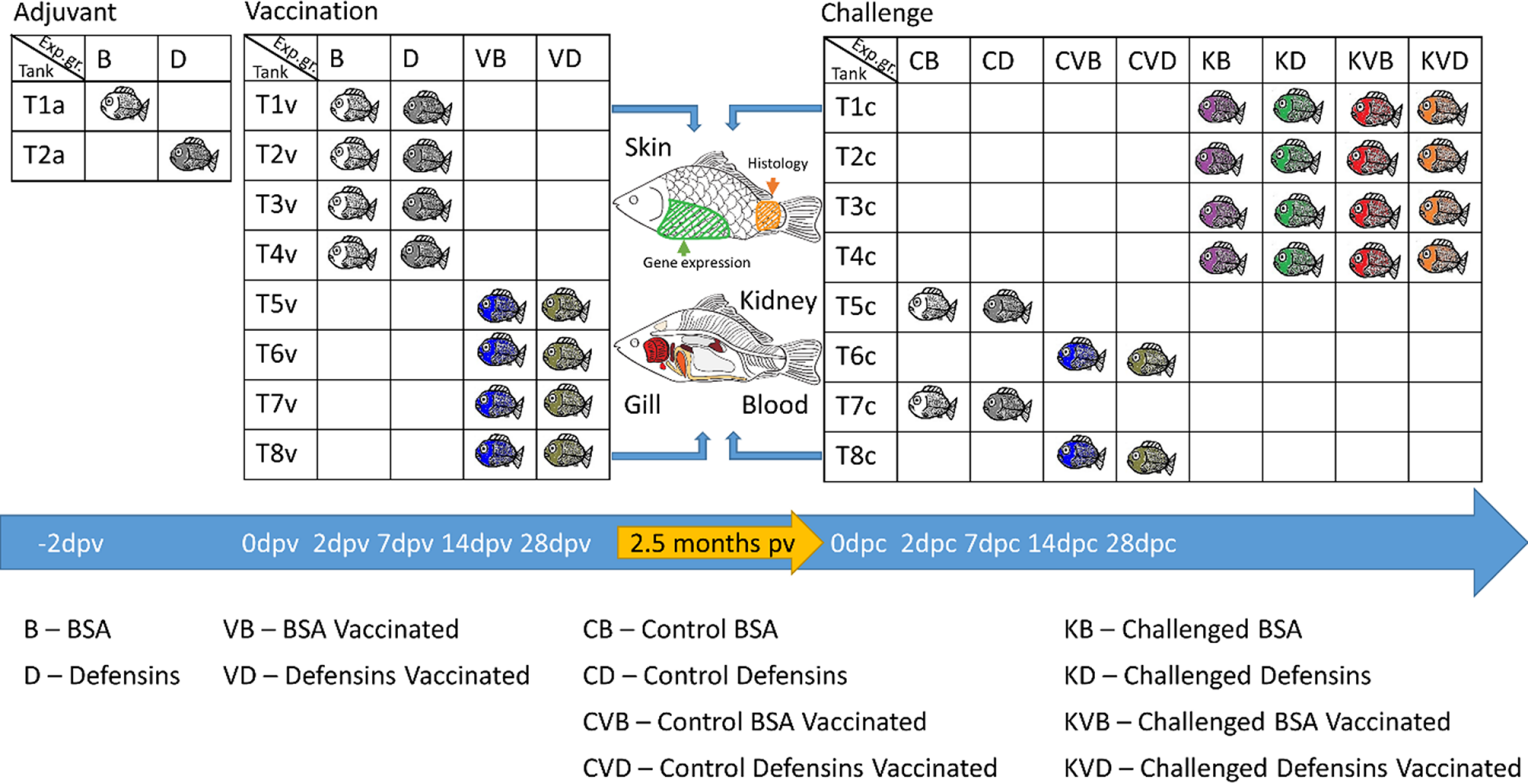
Schematic presentation of the tanks and experimental groups and samples collected from the fish used in this study.

For the vaccination experiment, fish were divided into eight 200 L tanks (**Figure 1**). Fish from four tanks (T1v-T4v; **Figure 1**) were exposed by bath treatment to tissue culture medium containing the vaccination virus KHV-T ΔDUT/TK (15) at a final concentration of 6.25×10^2^ plaque-forming units (PFU) mL^-1^. The bath treatment was administered in separate small plastic tanks with 40 L water at 23°C for 2h with constant aeration. The fish from four control tanks (T5v-T8v; **Figure 1**) were exposed to tissue culture medium without virus (mock vaccination). After vaccination/mock treatment, the fish were returned to the 200 L tanks and kept at a water temperature of 23 ± 1°C. Three tanks with vaccinated carp (T2v-T4v) and three tanks with mock vaccinated carp (T5v-T7v) were monitored daily for the development of clinical signs up to 28 days after vaccination. From one tank with mock vaccinated carp (T8v) and one tank with vaccinated carp (T1v), n = 5 fish treated with BSA or with defensins as adjuvants were collected at 2d, 7d, 14d and 28d post vaccination (dpv), euthanised by immersion in a bath with 0.5g L^-1^ Tricaine (MS222, Sigma) and blood and samples of the gill, skin and kidney were collected in RNA*later* (Sigma) and stored at -80°C until further analyses. Additional gill and skin samples from these carp were placed into 4% buffered formalin solution and used for histology. After a period of 28 days, the vaccinated fish were transferred to an 800 L tank and the non-vaccinated fish to a second 800 L tank and kept for another 1.5 months at 23 ± 1°C.

### Challenge Experiment

The challenge by an infection with a tissue culture derived, hyper-virulent wild type Polish isolate of CyHV-3 (19) was performed 75 dpv. For this, vaccinated and mock vaccinated carp were distributed to eight 200 L tanks according to the scheme presented in **Figure 1**. The fish in four tanks (T1c-T4c; **Figure 1**) were challenged with CyHV-3, while the fish in two tanks (T5c and T7c) served as unchallenged controls, and two tanks (T6c and T8c) served as unchallenged vaccination controls. Five tanks (T2c-T6c; **Figure 1**) were monitored for clinical signs, development and mortality. Additionally, one tank with CyHV-3 challenged fish (T1c), one tank with unchallenged control (T7c) and one tank with unchallenged vaccinated control (T8c) contained fish from which organs were collected for further analyses.

For CyHV-3 challenge, the fish from four tanks (T1c-T4c) with vaccinated and non-vaccinated carp were exposed to the Polish isolate of CyHV-3 at a final concentration of 1.4×10^3^ PFU mL^-1^ in small tanks with 40 L water at 23 ± 1°C for 2h with constant aeration (19). As controls, the fish from the additional four tanks (T5c-T8c) with vaccinated and with non-vaccinated carp were exposed to tissue culture medium without virus (mock challenge). After infection, the fish were returned to their 200 L tanks and kept at a water temperature of 23 ± 1°C for a period of 28 days. Humane endpoints were used for the fish which developed severe clinical signs and would not recover from infection. These fish were euthanised by immersion in a bath with 0.5g L^-1^ Tricaine. From each experimental group located in tanks T1c, T7c and T8c, n = 5 individuals were caught and euthanised with 0.5g L^-1^ Tricaine at 2d, 7d, 14d and 28d post challenge (dpc), blood and samples of the gill, skin and kidney were collected in RNA*later* and stored at -80°C until further analyses. Additional gill and skin samples were placed in 4% buffered formalin solution and used for histology. One tank each with mock vaccinated and with vaccinated carp was left as uninfected control and observed for the development of clinical signs and mortality as well.

### DNA Extraction

Genomic DNA was extracted from ~ 10 mg of tissue after mechanical lysis in a QIAgen Tissuelyser II (Qiagen), using the QIAamp DNA Mini Kit (Qiagen) in accordance with the manufacturer’s instructions. After isolation, the DNA samples were diluted to match the concentration of 50 ng μL^−1^ and stored at −80°C prior to qPCR analysis.

### RNA Extraction and cDNA Synthesis

Total RNA was extracted from tissue samples using Tri-Reagent (Sigma) in accordance with the manufacturer’s instructions. Any remaining genomic DNA was digested with 1 U of DNase I (Thermo Fisher Scientific) according to the manufacturer’s protocol. Synthesis of cDNA was performed from 300 ng of total RNA using 100 U of Maxima™ Reverse Transcriptase with random hexamers and oligo dT priming (Thermo Fisher Scientific). A non-reverse transcriptase control was included in the analysis of each sample. cDNA samples were diluted 1:40 with nuclease-free water (Thermo Fisher Scientific) prior to RT-qPCR analysis.

### qPCR/RT-qPCR

CyHV-3 DNA detection and quantification were performed using a probe-based real-time qPCR (TaqMan), detecting CyHV-3 ORF89 modified from the protocol developed by Gilad et al. (20), as described earlier by Adamek et al. (9). The results are presented as the total number of virus genome copies per 250 ng of DNA.

For quantifying viral and host mRNA, a SYBRGreen-based RT-qPCR was used. This method was used for measuring the expression of *cd4*, *cd8b1*, *igm* and *igz2* in skin and kidney as well as the skin and gill barrier markers *cdh1*, *ck15*, *cldn23*, *dsc2*, *def1b*, *muc2like*, *muc5b*, *ocldn* and *inos*. Also the bacterial load and expression of bacterial rRNA was measured using this method. Reactions were performed in duplicate using the Maxima SYBR Green 2× mastermix (Thermo Fisher Scientific) in a StepOnePlus real-time PCR machine (Thermo Fisher Scientific). The reaction mix was prepared as follows: 1× Maxima SYBR Green mastermix (with 100 nM of ROX), 0.2 μM of each primer (sequences in **Supplementary Table 1**), 5.0 μL of DNA (50 ng μL^−1^) or 20×diluted cDNA and nuclease-free water to a final volume of 25 μL. The amplification programme included an initial denaturation at 95°C for 10 min, followed by 40 cycles of denaturation at 95°C for 30 s, annealing at 55°C for 30 s and elongation at 72°C for 30 s. A dissociation curve was performed at the end of each run. For quantification, a recombinant DNA plasmid standard curve from 10^1^ to 10^7^ gene copies was prepared and used for quantifying the copy number from each sample as described by Adamek et al. (9).

For normalisation of the gene expression data measured by RT-qPCR, the gene encoding the 40S ribosomal protein S11 and the elongation factor 1 alpha were used as reference genes. The suitability of both genes as reference genes was confirmed by BestKeeper Software analysis (21) (**Supplementary Table 2**). The level of gene expression was shown as the copy number of the gene normalised against 1 × 10^5^ copies of the 40S ribosomal protein S11 and the elongation factor 1 alpha (normalised copy number, NCM) according to the following formula:


$$Normalisedcopynumber=mRNAcopiesperPCRfortargetgene/\left[\left(mRNAcopiesperPCRforreferencegene\;1+mRNAcopiesperPCRforreferencegene2\right)/2\times 10^{5}\right].$$

### Integrated Fluidic Circuit (IFC) Nanoscale RT-qPCR

The RNA from skin samples was extracted using 1 mL TRIzol Reagent (Thermo Fisher Scientific) and subsequently purified with the RNeasy Mini Kit (Qiagen). Residual DNA was digested with RNase-free DNase I for 12 min. The concentration and the purity of the isolated RNA were evaluated using the NanoDrop OneC spectrophotometer (NanoDrop Technologies). Agarose-gel electrophoresis validated the presence of intact 18S and 28S rRNA bands without genomic DNA contaminations.

The Pyrosequencing Assay Design software v.1.0.6 (Biotage) was used to design 48 carp-specific oligonucleotide primers (**Supplementary Table 3**). The efficiency of each primer pair was evaluated using quantitative PCR (qPCR) to establish primer pair-specific calibration curves based on a dilution series (10^3^ to 10^7^ copies) of the respective PCR-generated amplicons. These qPCR assays were conducted on the LightCycler 96 System (Roche), using the SensiFAST SYBR No-ROX Kit (Bioline) in clear LightCycler 480 multi-well plates (Roche).

The integrated fluidic circuit (IFC) technology of the Fluidigm Gene Expression biochips was used to profile the expression of 44 target genes (listed in **Supplementary Table 3**) and four reference genes (*actb*, *ef1a*, *gapdh*, *rps11*) in the extracted RNA from skin specimens (primer sequences in **Supplementary Table 3**). These multiplex analyses were performed on 48.48 Dynamic Array IFC chips (Fluidigm) using the BioMark HD system (Fluidigm). To this end, we adjusted total RNA at a concentration of 10 ng/µL and reverse-transcribed 1 µL (42°C, 30 min) using the Reverse Transcription Master Mix (Fluidigm). Then, the resulting cDNA aliquots were mixed with primers (100 µM) and the PreAmp master mix (Fluidigm) and individually preamplified in 13 cycles (95°C, 15 s; 60°C, 4 min) in a TAdvanced thermocycler (Biometra). After the pre-amplification step, exonuclease I (New England BioLabs) was added to degrade single-stranded oligonucleotide primers. After a 30-min incubation period at 37°C, 43 µL TE buffer (Sigma) were added per sample. Each 50-µL-cDNA sample was diluted in SsoFast EvaGreen Supermix with Low ROX (Bio-Rad) and 20×DNA Binding Dye Sample Loading Reagent (Fluidigm) to produce the sample mixes. After priming the 48.48-IFC chips in the MX Controller (Fluidigm), the primers and the sample mixes were transferred to the assay and sample inlets on the primed 48.48-IFC chip. Finally, multiplex quantitative PCR (qPCR) was conducted following the manufacturer’s thermal protocol ‘GE Fast 48x48 PCR+Melt v2.pcl’. The obtained qPCR data were analysed using the Fluidigm RealTime PCR Analysis Software v. 4.5.2 and normalised against the geometric mean of suitable normaliser genes (*actb*, *ef1a*, *rps11*) which were selected based on BestKeeper Software analysis (**Supplementary Table 2**).

### Histology

Skin and gill samples fixed in 4% buffered formalin were subsequently dehydrated in a series of graded ethanol and embedded into paraffin wax in accordance with a standard laboratory protocol. From paraffin blocks, sections were cut to a thickness of 3 μm and stained with Alcian Blue Periodic Acid Schiff (AB-PAS). To evaluate the impact of the infection on the morphology of skin and branchial tissue, the following changes were recorded: (i) presence or absence of skin epithelium with mucus filled goblet cells and (ii) in gills, proliferation of the intra-lamellar cellular mass and the thickness of the secondary lamella.

### Serum Neutralisation Test and Blood Sodium Concentration

Immediately after collection, blood samples were centrifuged at 600 × g at 4°C for 15 min, the supernatant serum was collected and instantly frozen at -80°C. The serum neutralisation test (SNT) was performed as described earlier (22) with minor modifications. Briefly: each serum sample was divided into three 10 µL aliquots and heat inactivated at 45°C for 30 min in an Eppendorf 5331 Mastercycler Gradient thermocycler. Afterwards, 190 µL of cell culture medium for KFC cells (EMEM, 10% FCS, 1 × NEAA, 10 IU/mL penicillin and 100 mg/mL streptomycin, Sigma) was added and the serum was diluted in two-fold serial dilution steps in a 96-well plate (Nunc, ThermoFisher Scientific). For two aliquots of serum, 100 µL of dilutions from 1/20 to 1/2560 were used for inactivation of 100 µL CyHV-3 (1 × 10^3^/mL TCID^50^; a Polish isolate, which was used for the challenge). This resulted in final dilutions of the serum from 1/40 to 1/5120. To test for any cytotoxic effects of the serum, the third aliquot was mixed with 100 µL of cell culture medium for KFC cells. The serum/virus or serum/medium mixes were subsequently incubated at 4°C for 24h and then transferred to the wells of a 96-well plate with monolayers of KFC cells and incubated at 20°C. The cell monolayers were observed for the formation of a cytopathic effect in wells inoculated with serum/virus mixes and for a cytotoxic effect in wells with the serum/medium mixes for 14 days.

Blood sodium levels were determined in serum samples with a flame photometer (Bayer Diagnostics) as described earlier (23).

### Statistical Analysis

SigmaPlot 12.5 software (Systat Software) was used for statistical analysis. Normalised data on gene expression and virus load were transformed using a Log10(x) transformation before further statistical analysis. Significant differences (*p* ≤ 0.05) in virus and bacteria load and in gene expression, mortality, SNT and sodium level were assessed using a one-way or two-way ANOVA, with subsequent pairwise multiple comparisons using the Holm-Sidak method. Data are presented as box plots of 25-75 percentiles (± minimum and maximum values) with an indication of all points and the median using Prism 9 software (GraphPad Software).

## Results

### Response of Carp to Adjuvant Administration and to Vaccination

**Figure 2:**
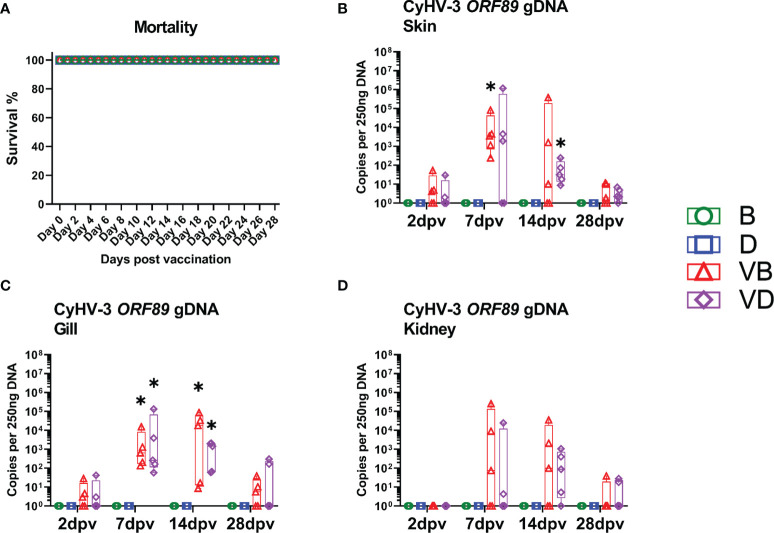
Mortality and virus load after the vaccination with KHV-T ΔDUT/TK. **(A)** Approximated mortality during vaccination. **(B)** Virus load in the skin collected 2 days post vaccination (dpv), 7 dpv, 14 dpv, 28 dpv. **(C)** Virus load in the gills. **(D)** Virus load in the kidney. The description of experimental groups: B – BSA non-vaccinated, D – defensins non-vaccinated, VB - BSA vaccinated, VD – defensins vaccinated. The results are presented as 25%-75% box plots with min. and max. values as whiskers with indication of all data points. The data are shown as virus genome copy per 250 ng of extracted DNA. * indicates statistical significant difference to non-vaccinated controls at *p* < 0.05. Statistical analysis was performed with two-way ANOVA with multiple comparisons test performed with the Holm-Sidak method.

Bath treatment of carp in a solution with carp defensins as vaccination adjuvants did not evoke any behavioural or visible clinical changes in subjected carp. Also, upon infection of carp with the live attenuated KHV-T ΔDUT/TK vaccinate virus, no obviously visible behavioural changes were noticed, nor were macroscopically visible clinical signs recorded. The vaccinated carp had a minor reduction in feed uptake around days 7-10 post vaccination (dpv). No mortality was recorded (**Figure 2A**). For observing the progress of the infection of carp with the KHV-T ΔDUT/TK, the virus load was monitored in the skin, gills and kidney (**Figures 2B–D**). The virus was detectable in all tested tissues of the selected fish, but the load varied largely and peaked at 14 dpv for the BSA group, and 7 dpv for the defensin group, with only two fish over 100,000 copies per 250 ng of DNA. The virus could be detected in gills of all fish at days 7 and 14 post vaccination, while the skin or kidney of some fish remained negative at these time points (**Figures 2B, D**). Furthermore, most of the fish had a virus load below 10,000 copies per 250ng of DNA in all tissues (**Figures 2B–D**), which is considered to be a subclinical virus burden. The viral mRNA could be detected only in some fish at 7 and 14 dpv and remained on a very low level, <100 normalised copies in the skin and <10 copies in the kidney (**Supplementary Figures 1A, B**). The vaccination also did not lead to a significant drop in the blood sodium level at 7 dpv. This could be an indication that the vaccinated carp did not develop a KHVD-related disruption of their osmotic balance (**Supplementary Figure 2A**). Measuring the changes in the total bacteria content (expression of 16S rRNA) showed that defensin-treated fish had statistically significantly lower (66-fold) expression of bacteria 16S, while vaccinated fish in the defensin group had 88-fold increased expression of bacteria 16S, which could be construed as a disbalancing effect of defensins on bacteria flora. Nonetheless, the vaccination induced only minor changes in the amount of bacteria, which is not an indicator of disruption of the mucosal barrier (**Supplementary Figure 2B**).

**Figure 3:**
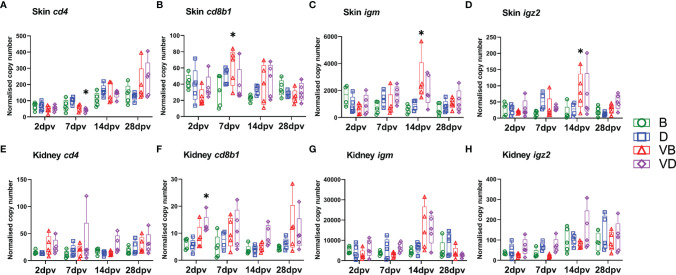
Levels of transcripts coding for proteins involved in immune responses to vaccination with KHV-T ΔDUT/TK, measured with RT-qPCR in skin: **(A)** *cd4*, **(B)** *cd8b1*, **(C)** *igm*, **(D)** *igz2*, and kidney: **(E)** *cd4*, **(F)** *cd8b1*, **(G)** *igm*, **(H)** *igz2*. The description of experimental groups: B – BSA non-vaccinated, D – defensins non-vaccinated, VB - BSA vaccinated, VD – defensins vaccinated. The results are presented as 25%-75% box plots with min. and max. values as whiskers with indication of all data points. The data are shown as normalised copy numbers. * indicates statistical significant difference to non-vaccinated control at *p* < 0.05. Statistical analysis was performed with two-way ANOVA with multiple comparisons test performed with the Holm-Sidak method.

In order to monitor the induction of immune responses in carp infected with the KHV-T ΔDUT/TK virus, the transcription of the immune genes *cd4*, *cd8b1*, *igm* and *igz* was analysed in the skin (**Figures 3A–D**) and kidney (**Figures 3E–H**) by RT-qPCR. In the skin, the vaccination induced only minor immune responses. Compared to uninfected carp, in the group of carp vaccinated and treated with defensins as adjuvants, only the expression of *cd4* was significantly downregulated two-fold at 7 dpv (**Figure 3A**). In the carp vaccinated and treated with BSA as mock-adjuvant, the transcription of *cd8b1* was upregulated two-fold at 7 dpv (**Figure 3B**) and the expression of *igm* and *igz2* was significantly upregulated five-fold at 14 dpv (**Figures 3C, D**). The responses in the kidney were even lower; only the transcription of *cd8b1* was statistically significantly upregulated two-fold in vaccinated carp (**Figure 3F**). The strongest effect of the vaccination was recorded when the virus neutralisation activity of blood serum was measured, which rose at 28 dpv to a statistically significantly higher dilution level (1/736) compared to the controls (1/20-1/32) (**Table 1**). The vaccination virus did not cause any clinical signs related to KHVD and induced some T and B cell responses. The vaccination also induced production of the virus-neutralising antibodies. The use of a mixture of beta-defensin 1, 2 and 3 as adjuvants did not boost immune responses when compared with carp treated with BSA as adjuvant control. However, they could have some effect on the bacteria community of the skin.

**Table 1:** Serum neutralisation test after vaccination with KHV-T ΔDUT/TK.

SNT	Experimental group			
Time point	B	D	VB	VD
2 dpv	32 ± 11	20 ± 0	20 ± 0	20 ± 0
7 dpv	24 ± 9	24 ± 9	80 ± 73	56 ± 59
14 dpv	24 ± 9	24 ± 9	64 ± 59	56 ± 59
28 dpv	20 ± 0	28 ± 11	736 ± 526*	1256 ± 2172

The description of experimental groups: B – BSA non-vaccinated, D – defensins non-vaccinated, VB - BSA vaccinated, VD – defensins vaccinated. The results are presented as a mean ± SD of the highest serum dilution inactivating the CyHV-3. * indicates statistical significant difference when compared with the control at p < 0.05. Statistical analysis was performed with two-way ANOVA with multiple comparisons test performed with the Holm-Sidak method.

### Response to a Challenge Infection

**Figure 4:**
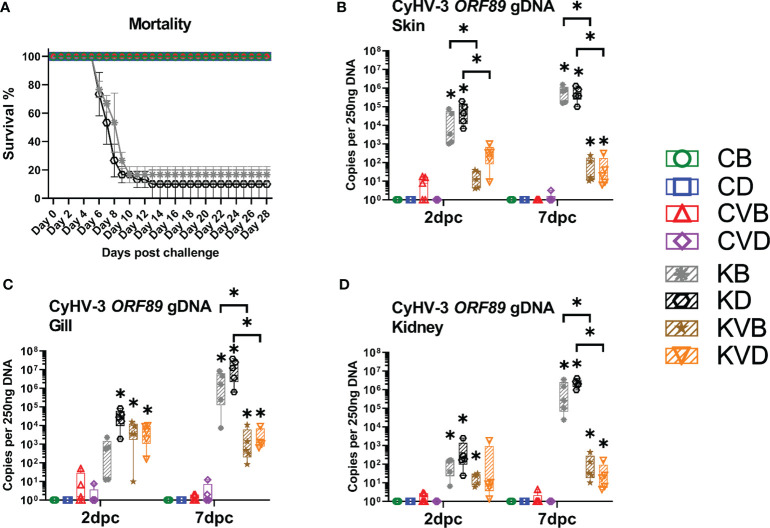
Mortality and virus load during the challenge with CyHV-3. **(A)** Approximated mortality during challenge. **(B)** Virus load in the skin collected 2 dpc and 7 dpc. **(C)** Virus load in the gills. **(D)** Virus load in the kidney. The description of experimental groups: CB – BSA, non-vaccinated, unchallenged control, CD – defensins, non-vaccinated, unchallenged control, CVB – BSA, vaccinated, unchallenged control, CVD – defensins, vaccinated, unchallenged control, KB - BSA non-vaccinated, CyHV-3 challenged, KD – defensins non-vaccinated, CyHV-3 challenged, KVB – BSA, vaccinated, CyHV-3 challenged, KVD – defensins, vaccinated, CyHV-3 challenged. The results are presented as 25%-75% box plots with min. and max. values as whiskers with indication of all data points. The data are shown as the virus genome copy numbers per 250 ng of extracted DNA. * indicates statistical significant difference at *p* < 0.05. Statistical analysis was performed with two-way ANOVA with multiple comparisons test performed with the Holm-Sidak method.

A challenge infection of vaccinated and non-vaccinated control carp with a hyper-virulent wild type CyHV-3 variant induced a severe disease with 80-100% mortality in non-vaccinated fish, while in vaccinated fish, no mortality was recorded (**Figure 4A** and **Supplementary Figures 3A–F**) and the vaccinated fish developed no clinical signs of a CyHV-3 infection despite being kept together with non-vaccinated fish in the same tanks. In non-vaccinated carp, mortality, which was typical for the course of a CyHV-3 infection, started by 7 dpc and ended at 13 dpc (**Figure 4A**). When the virus load was considered, significant differences could be observed between vaccinated and non-vaccinated carp. Skin samples from non-vaccinated challenged carp harboured a high virus load already 2 dpc (mean 24,639 to 71,171 CyHV-3 specific DNA copies per 250 ng of DNA), while in skin samples from vaccinated carp, a low virus load of <1,000 CyHV-3 specific DNA copies was observed. In vaccinated fish, the viral load remained on this level also at 7 dpc, whereas in non-vaccinated carp a mean of 602,622 to 662,255 copies was reached per 250 ng of DNA. This meant over 1,000-fold reduction in virus load in vaccinated fish (**Figure 4B**). Also, in gills, the virus load at 7 dpc was >1,000-fold lower in vaccinated carp when compared with non-vaccinated carp (**Figure 4C**). Interestingly, the kidney of vaccinated carp exhibited some virus load at both time points (<1,000 copies of CyHV-3-specific DNA) which was an over 50,000-fold reduction when compared with the virus load in non-vaccinated fish of which the kidney harboured >1,000,000 copies of CyHV-3-specific DNA (**Figure 4D**). Additional qPCR for ORF55 fragment which was deleted in the vaccination virus showed that wild type virus was in majority (>99.9% of viral particles) in the tissues of challenged fish (**Supplementary Figures 4A–C**). The expression of viral mRNA encoding for ORF89 was highly elevated in non-vaccinated CyHV-3 challenged fish and nearly not detectable in the vaccinated, CyHV-3 challenged fish (**Supplementary Figures 4D–F**). This means that the virus in both vaccinated and non-vaccinated fish led to systemic infection; however, the pathogen load was highly reduced by vaccination.

**Figure 5:**
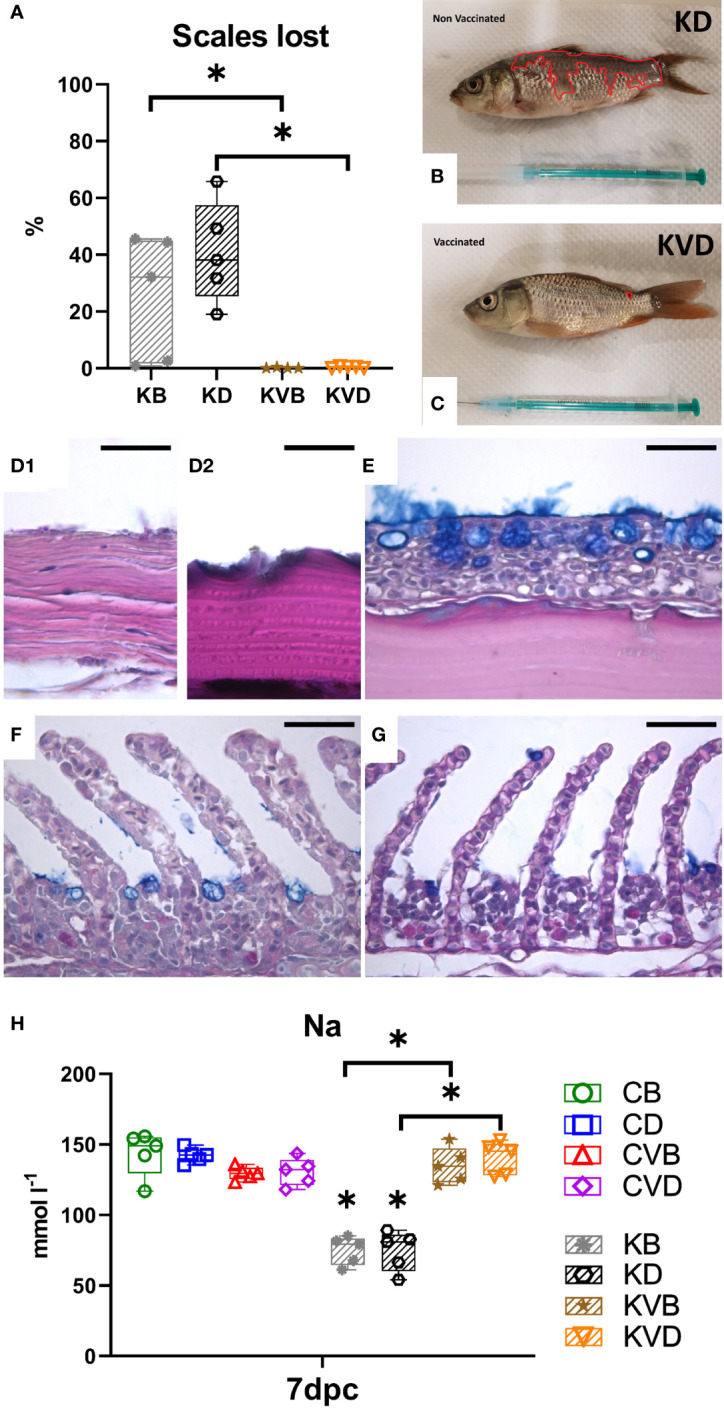
Macroscopic, pathohistological and pathophysiological effect of the CyHV-3 challenge at 7 dpc. **(A)** Percentage of the body integument where scales were lost during the challenge. The surface area of **(B)** non-vaccinated and **(C)** vaccinated fish which lost scales are outlined in red. **(D_1_)** Histology of the skin of non-vaccinated, CyHV-3 challenged fish which lost scales. **(D_2_)** Histology of the skin of non-vaccinated, CyHV-3 challenged fish which had intact scales but lost the epithelium. **(E)** Histology of the skin of vaccinated fish showing scales covered with epithelium. **(F)** Histology of the gills of non-vaccinated, CyHV-3 challenged fish showing thickening of secondary lamella and some increase in cell proliferation in the intralammellar spaces. **(G)** Histology of the gills of vaccinated, CyHV-3 challenged fish showing secondary lamella of normal thickness and some increase in cell proliferation in the intralammellar spaces. **(H)** Sodium level in blood serum. The description of experimental groups: CB – BSA, non-vaccinated, unchallenged control, CD – defensins, non-vaccinated, unchallenged control, CVB – BSA, vaccinated, unchallenged control, CVD – defensins, vaccinated, unchallenged control, KB - BSA non-vaccinated, CyHV-3 challenged, KD – defensins non-vaccinated, CyHV-3 challenged, KVB – BSA, vaccinated, CyHV-3 challenged, KVD – defensins, vaccinated, CyHV-3 challenged. The results are presented as 25%-75% box plots with min. and max. values as whiskers with indication of all data points. * indicates statistical significant difference at *p* < 0.05. Statistical analysis was performed with one-way ANOVA with the multiple comparisons test performed with Holm-Sidak method.

These challenged non-vaccinated fish developed severe, macroscopically visible changes on the skin with a progressing loss of scales, this reaching an average expansion over 25% of the body in non-vaccinated challenged fish treated with BSA as mock adjuvant, and over 41% in the defensins group (**Figures 5A, B**), while in vaccinated fish, single scales were missing (**Figures 5A, C**). The histological analysis revealed strongest pathological changes in the skin with a complete destruction of the skin epithelium in areas which had lost scales (**Figure 5D**_1_). The epithelium was also missing in parts where scales were present (**Figure 5D**_2_). The goblet cells disappeared together with the epithelium and therefore no mucus was present on the skin (**Figure 5D**_1_, D_2_). In vaccinated fish, no significant pathological alterations were observed in the skin and normal epithelium with goblet cells and mucus layer was present (**Figure 5E**). The gills of vaccinated and non-vaccinated fish showed no macroscopical changes; in histology, the gills of non-vaccinated fish showed an increased occlusion of the interlamellar space between secondary lamellae. Thickening or swelling of the branchial epithelium cells in secondary lamellae was noticed, and the goblet cells were very often empty (**Figure 5F**). In gills of vaccinated fish, some occlusion of the interlamellar was present but no swelling of secondary lamellae occurred (**Figure 5G**).

The pathological changes in the skin and gills of non-vaccinated carp caused by challenge infection with CyHV-3 led to a significant drop in blood sodium levels at 7 dpc (**Figure 5H**). The mean level of sodium dropped to 75 mmol/L, irrespective of whether the carp had been treated with defensins as adjuvants or with BSA. This sodium level was statistically significantly different to the level in the blood of vaccinated challenged fish, which had a mean sodium level of 135-140 mmol/L (**Figure 5G**). This was not different from the sodium level in the blood of non-treated controls and of vaccinated controls with a mean level of about 140 mmol/L. This shows that the vaccination protected from osmotic disbalance related with a CyHV-3 infection.

The vaccination effect could be noticed in virus neutralisation activity of blood serum, which was still statistically significantly elevated and remained at the dilution level of 1/600 to 1/2 688 in only vaccinated and in vaccinated CyHV-3 challenged fish at 2, 7, 14 and 28 dpc. In contrast, in non-vaccinated controls, the dilution level remained at <1/40 and was elevated by the CyHV-3 infection to the dilution level of <1/156 in CyHV-3 challenged non-vaccinated fish (**Table 2**). This strongly suggests that CyHV-3 neutralising antibodies contributed to the protection of vaccinated fish from developing a high virus load and clinical signs of KHVD.

**Table 2:** Serum neutralisation test after the CyHV-3 challenge.

SNT	Experimental group							
Time point	CB	CD	CVB	CVD	KB	KD	KVB	KVD
2 dpc	40 ± 24	24 ± 9	1952 ± 1987*	1440 ± 2104*	152 ± 155	156 ± 151	1024 ± 251*	1504 ± 2066*
7 dpc	20 ± 0	36 ± 26	968 ± 1003*	2688 ± 2401*	80 ± 49	56 ± 59	928 ± 511*	1472 ± 1052*
14 dpc	21 ± 0	20 ± 0	608 ± 429*	640 ± 1078*	§	§	1728 ± 2130*	1856 ± 2029*
28 dpc	22 ± 0	20 ± 0	1792 ± 2081*	704 ± 351*	§	§	1920 ± 1810*	2368 ± 1805*

The description of experimental groups: CB – BSA, non-vaccinated, unchallenged control, CD – defensins, non-vaccinated, unchallenged control, CVB – BSA, vaccinated, unchallenged control, CVD – defensins, vaccinated, unchallenged control, KB - BSA non-vaccinated, CyHV-3 challenged, KD – defensins non-vaccinated, CyHV-3 challenged, KVB – BSA, vaccinated, CyHV-3 challenged, KVD – defensins, vaccinated, CyHV-3 challenged. The results are presented as a mean ± SD of the highest serum dilution inactivating the CyHV-3. § indicates that fish from these experimental groups had to be removed before the time point due to pathological changes.* indicates statistical significant difference when compared with the control at p < 0.05. Statistical analysis was performed with two-way ANOVA with multiple comparisons test performed with the Holm-Sidak method.

### Immune Responses in Skin

**Figure 6:**
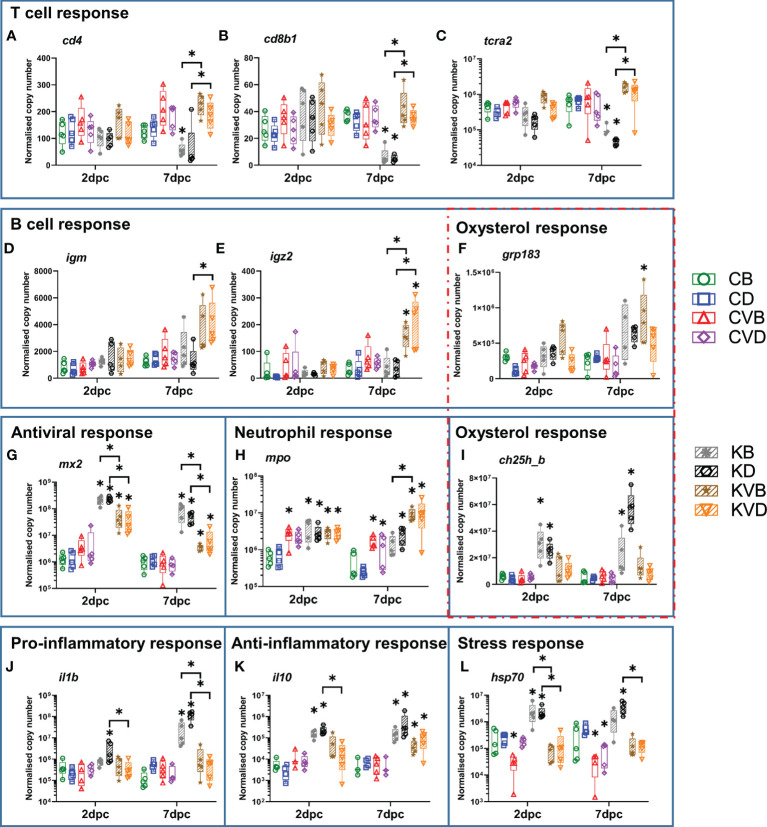
Level of transcripts coding for proteins involved in immune responses after the CyHV-3 challenge at 2 dpc and 7 dpc measured with classical RT-qPCR and integrated fluidic circuit nanoscale RT-qPCR in skin. Presented are selected markers for T cell response: **(A)** *cd4*, **(B)** *cd8b1*, **(C)** *tcra2*, B cell response: **(D)** *igm*, **(E)** *igz2*, **(F)** *grp183* which is also involved in oxysterol response, antiviral response **(G)** *mx2*, neutrophil-specific response: **(H)** *mpo*, oxysterol response: **(I)** *ch25h_b*, pro-inflammatory response: **(J)** *il1b*, anti-inflammatory response: **(K)** *il10*, stress response: **(L)** *hsp70.*The description of experimental groups: CB – BSA, non-vaccinated, unchallenged control, CD – defensins, non-vaccinated, unchallenged control, CVB – BSA, vaccinated, unchallenged control, CVD – defensins, vaccinated, unchallenged control, KB - BSA non-vaccinated, CyHV-3 challenged, KD – defensins non-vaccinated, CyHV-3 challenged, KVB – BSA, vaccinated, CyHV-3 challenged, KVD – defensins, vaccinated, CyHV-3 challenged. The results are presented as 25%-75% box plots with min. and max. values as whiskers with indication of all data points. The data are shown as normalised copy numbers. * indicates statistical significant difference at *p* < 0.05. Statistical analysis was performed with two-way ANOVA with multiple comparisons test performed with the Holm-Sidak method.

In non-vaccinated carp, the challenge infection with CyHV-3 led to pronounce changes in the expression of the immune genes in the skin (**Figures 6A–L** and **Supplementary Figures 5A–N**) Generally, profound hallmarks of immunosuppression of T cell responses could be noticed in non-vaccinated carp at 7 pdc. The mRNA expression of *cd4* was two-fold and c*d8b1* was six-fold downregulated and *trca2* five- to 14-fold in the skin of non-vaccinated, challenged fish at 7 dpc (**Figures 6A–C**). At this time point, the five- to 50-fold lower *cd8a1* transcript levels (**Supplementary Figure 5F**) were concomitant with the four- to 17-fold reduced *cd8b2* levels in the BSA and defensin groups, respectively (**Supplementary Figure 5G**). In contrast to this, in vaccinated carp under challenge infection, both *cd8a* genes were >three-fold upregulated to a level, which resulted in a statistically significant difference between challenged vaccinated and non-vaccinated fish. In addition, the transcription of *tcra1* was downregulated four- to 31-fold (**Supplementary Figure 5E**), in non-vaccinated carp. Similar to the transcription of *cd8a1* and *cd8b2*, the transcription of *tcra1* and *a2* was >three-fold upregulated in vaccinated carp. The expression of *igm* was elevated in vaccinated fish but the difference was not statistically significant when compared with controls. Nonetheless, in the skin of vaccinated fish after challenge infection, the level was statistically significantly higher than in non-vaccinated challenged fish, namely two- to three-fold at 7 dpc (**Figure 6D**). The expression of *igz2* was boosted two- to four-fold in the vaccinated challenged fish at 7 dpc (**Figure 6E**). The levels were four- to five-fold higher in vaccinated challenged fish than in non-vaccinated challenged fish (**Figure 6E**).

In the skin of non-vaccinated carp, inflammatory responses were increased under challenge infection with the wild type CyHV-3 when compared with the controls and challenged vaccinated fish. The expression of *il1b* was upregulated 15-fold already at 2 dpc in carp treated with defensins and challenged with CyHV-3 and this upregulation increased to 189-fold in carp in the BSA treated group and to 215-fold in carp in the defensin group at 7 dpc (**Figure 6J**). The expression of the anti-inflammatory *il10* was also upregulated up to 78-fold 2 dpc and 7 dpc in the non-vaccinated challenged fish previously treated with BSA or defensins as adjuvants (**Figure 6K**). Interestingly, the expression of *il10* was also upregulated six- to 17-fold in vaccinated challenged fish in the absence of *il1b* upregulation, which suggests that vaccination prior to CyHV-3 exposure reinforced humoral immune response by inducing expression of this cytokine (**Figures 6J, K**).

The infection with CyHV-3 increased the transcript levels of apoptosis-related genes in the skin of non-vaccinated carp, hallmarked by a two- to 13-fold upregulation of *apaf1*, *casp9* and *iap* at 2 dpc and *p53* at 7 dpc (**Supplementary Figures 5A, C,–E**). In contrast to this, the transcription of *casp6* was four- to 10-fold downregulated at 7 dpc (**Supplementary Figure 5B**). In the skin of vaccinated challenged fish, no significant changes in the expression of apoptotic genes were observed.

A robust antiviral response was induced in the skin of both non-vaccinated and vaccinated challenged carp. The transcription of *mx2* was upregulated >160-fold (**Figure 6G**). The transcription of *trim21* and *gig1* underwent a >1,000-fold upregulation already at 2 dpc (**Supplementary Figures 5J, M**). The transcription of *ifna2* and *vig1* was upregulated >300 and >600-fold in the skin of non-vaccinated carp challenged with a CyVH-3 (**Supplementary Figures 5K, N**). In the skin of vaccinated carp, a 10-fold lower antiviral response was measured compared to the response in the skin of non-vaccinated carp. However, this antiviral response was still statistically significant when compared with vaccinated and non-vaccinated, non-challenged controls. The transcription of *irf7* was upregulated to the lowest extent, up to 45-fold (**Supplementary Figure 5L**).

Only a part of the cholesterol/oxysterol pathway was regulated in response to the infection, with a strong upregulation of the transcription of *ch25h_b* four- to 12-fold, both 2 dpc and 7 dpc in non-vaccinated carp challenged with CyHV-3 when compared to non-infected and to vaccinated and CyHV-3-challenged carp (**Figure 6I**). The transcription of *ch25h_a* was not regulated in infected carp nor was *fdps*, which is responsible for cholesterol synthesis, and *cyp7b1*, which hydroxylates 25-hydroxycholesteron to 7a,25-dihydroxycholesterol (**Supplementary Figures 6A–H**). The expression of *grp183*, which binds 7a,25-diHC ligand for direct positioning of B cells during humoral immune responses, was significantly upregulated three-fold in vaccinated challenged carp compared with non-infected and non-vaccinated, CyHV-3-infected fish (**Figure 6F**).

The transcription of the antimicrobial agent *fel* was slightly (<four-fold) down regulated in non-vaccinated challenged fish compared to non-infected carp, while it was upregulated three-fold in the vaccinated challenged fish at 7 dpc (**Supplementary Figure 5I**).

The expression of genes related to stress responses was increased in the skin of carp under challenge infection with CyHV-3. The expression of *hsp70* was upregulated nine- to 10-fold at 2 dpc and five- to seven-fold at 7 dpc in non-vaccinated challenged fish, while in vaccinated challenged fish, the expression of *hsp70* was not statistically significantly upregulated (**Figure 6L**). The expression of the neutrophil marker *mpo* was boosted by vaccination and by challenge infection four- to 10-fold at 2 dpc and 7 dpc (**Figure 6H**). At 7 dpc, it was further statistically significantly upregulated four- to six-fold in the skin of vaccinated carp under challenge infection (**Figure 6H**). The expression of several additional genes (*bf/c2*, *crp2*, *il12p35*, *pkz* and *serpinb1*) was not regulated upon infection (**Supplementary Figures 6A–H**).

### Immune Responses in Kidney

Also in the kidney an immunosuppression of T cell responses was noticed in non-vaccinated carp responding to the CyHV-3 challenge. This tissue was used to relate the systemic responses to the skin responses. The kidney responded with a four- to five-fold downregulation of the *cd4* expression at 7 dpc (**Supplementary Figure 7A**). The expression of *cd8b1* was three-fold upregulated in vaccinated challenged fish treated with BSA as mock adjuvant at 2 dpc (**Supplementary Figure 7B**). Furthermore, these expression levels were three-fold higher in vaccinated challenged fish when compared with challenged non-vaccinated fish (**Supplementary Figure 7B**). No statistically significant changes in expression of *igm* and *igz2* were detected (**Supplementary Figures 7C, D**).

### Disruption of Barrier Function of Skin and Gills

**Figure 7:**
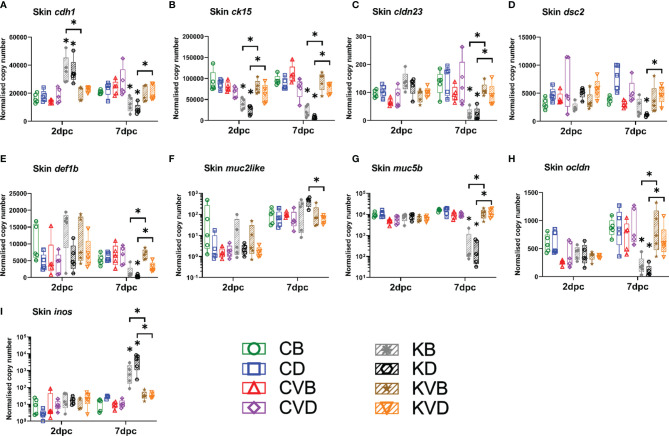
Level of transcripts coding for proteins involved in skin barrier integrity and function after the CyHV-3 challenge at 2 dpc and 7 dpc measured with RT-qPCR: **(A)** *cdh1*, **(B)** *ck15*, **(C)** *cldn23*, **(D)** *dsc2*, **(E)** *def1b*, **(F)** *muc2like*, **(G)** *muc5b*, **(H)** *ocldn*, **(I)** *inos*. The description of experimental groups: CB – BSA, non-vaccinated, unchallenged control, CD – defensins, non-vaccinated, unchallenged control, CVB – BSA, vaccinated, unchallenged control, CVD – defensins, vaccinated, unchallenged control, KB - BSA non-vaccinated, CyHV-3 challenged, KD – defensins non-vaccinated, CyHV-3 challenged, KVB – BSA, vaccinated, CyHV-3 challenged, KVD – defensins, vaccinated, CyHV-3 challenged. The results are presented as 25%-75% box plots with min. and max. values as whiskers with indication of all data points. The data are shown as normalised copy numbers. * indicates statistical significant difference at *p* < 0.05. Statistical analysis was performed with two-way ANOVA with multiple comparisons test performed with the Holm-Sidak method.

The severe macroscopic and microscopic changes in skin combined with increased inflammation could affect the barrier function of skin. However, differently to the histopathological evaluation, the gene expression was investigated in parts of the skin with no, or very minor macroscopical changes. Still, a large impact of the infection could be detected on the skin barrier at molecular level (**Figures 7A–I**). The vaccination however offered protection against these changes. Indication of a disruption of adherence junctions between epithelial cells were noticed with a two- to three-fold downregulation of the expression of *cdh1* only in non-vaccinated CyHV-3-challenged fish, while vaccinated carp under challenge infection had mRNA levels similar to those in the control fish (**Figure 7A**). Similar differences between vaccinated and non-vaccinated fish challenged with CyHV-3 were seen in the expression of tight junctions markers *cldn23*, which was downregulated six- to seven-fold (**Figure 7C**), and *ocldn*, which was downregulated four- to nine-fold (**Figure 7H**). Similarly, seven-fold downregulation was recorded for desmosome marker *dsc2* (**Figure 7D**). The expression of gene *ck15* encoding cytokeratin 15, a marker for epithelial cells, was downregulated in non-vaccinated, CyHV-3 challenged fish two- to four-fold already at 2 dpc and the downregulation increased to five- to 12-fold at 7 dpc (**Figure 7B**). In the skin of vaccinated, CyHV-3 challenged fish, these changes were not recorded. The vaccination also offered protection against a CyHV-3 infection-induced drop in expression of the gene *defb1* encoding defensin beta 1 three- to 17-fold (**Figure 7E**), and the gene encoding mucin 5b, which was downregulated 30- to 54-fold (**Figure 7G**) after challenge of non-vaccinated fish. In contrast to this, the expression of the *muc2like* gene encoding a secreted mucin was upregulated (**Figure 7F**), which coincided with 86- to 163-fold increased expression of *inos* (**Figure 7I**), being a marker of inflammatory responses. While pathological effects were increased in the carp which had been treated with defensins as adjuvant prior to infection, the mucosal integrity markers in challenged vaccinated fish from both groups were not affected. This means that vaccination protected against disruption of skin barrier.

In addition to skin, the impact of the infection on the barrier of gills was also investigated. The highest changes to the skin barrier occurred 7 dpc. Hence, the effect of the infection on the gills was measured at this time point. The effect of the infection on the gills was lower than on the skin (**Supplementary Figures 8A–I**). The infection mainly affected the expression of *ck15* (encoding cytokeratin 15), whose expression was downregulated seven-fold in both CyHV-3 challenged groups (**Supplementary Figure 8B**), which coincided with a 32- and 49-fold increase in the expression of *inos* (**Supplementary Figure 8I**), indicating inflammation. The vaccination, however, prevented changes in the expression of these genes under challenge infection with a virulent virus variant. The infection also significantly downregulated the expression of *dsc2* when compared to the controls (**Supplementary Figure 8E**) and the expression of *muc5b* when compared to challenged vaccinated fish (**Supplementary Figure 8G**). There were some additional genes, which were significantly regulated by vaccination and the challenge infection, but the change in expression was less than two-fold. This included *ocldn* (**Figure Supplementary 8F**) and *cdh1* (**Supplementary Figure 8A**). As the pathological effect of the infection on the gill barrier was lower, the protection induced by the vaccination was also less prominent, but nonetheless still prevented the drop in epithelial skin markers and an increase in inflammation.

### Secondary Bacterial Infections (SBIs)

**Figure 8:**
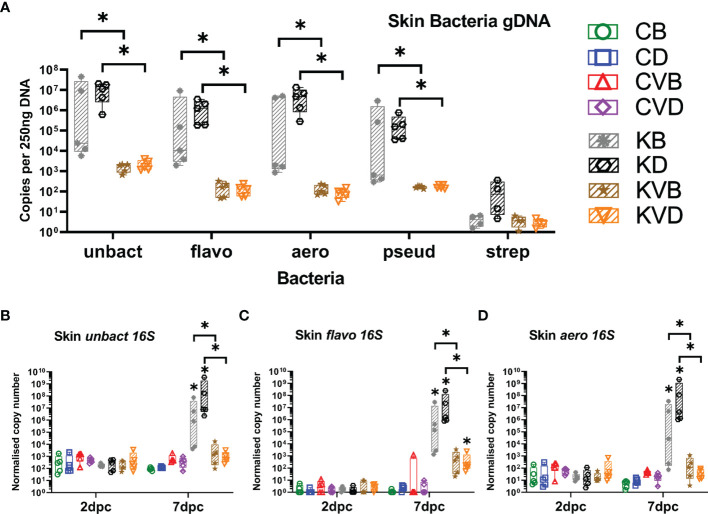
Bacterial load and level of 16S rRNA for selected bacteria species after challenge with CyHV-3 measured with qPCR or RT-qPCR. **(A)** All bacteria (unbact), flavobacteria (flavo), aeromonads (aero), pseudomonads (pseud) and streptococci (strep) load in skin at 7 dpc. Expression of bacterial 16S rRNA: **(B)** all bacteria 16S in skin at 2 dpc and 7 dpc, **(C)** flavobacteria 16S in skin at 2 dpc and 7 dpc, **(D)** aeromonas 16S in skin at 2 dpc and 7 dpc. The description of experimental groups: CB – BSA, non-vaccinated, unchallenged control, CD – defensins, non-vaccinated, unchallenged control, CVB – BSA, vaccinated, unchallenged control, CVD – defensins, vaccinated, unchallenged control, KB - BSA non-vaccinated, CyHV-3 challenged, KD – defensins non-vaccinated, CyHV-3 challenged, KVB – BSA, vaccinated, CyHV-3 challenged, KVD – defensins, vaccinated, CyHV-3 challenged. The results are presented as 25%-75% box plots with min. and max. values as whiskers with indication of all data points. Bacteria load data are shown as genomic 16S copy numbers normalised for 250 ng of extracted DNA. The expression data are shown as normalised copy numbers. * indicates statistical significant difference at *p* < 0.05. Statistical analysis was performed with two-way ANOVA with multiple comparisons test performed with the Holm-Sidak method.

The disruption of the elements of skin and gill barrier could lead to an uncontrolled increase in the load of skin bacteria. Therefore, the differences in bacterial load between vaccinated and non-vaccinated carp were evaluated. Using genomic DNA, much higher changes in the number of bacteria were recorded in the skin than in gills (**Figure 8A** and **Supplementary Figure 9A**). For example, when the entire bacteria flora was quantified with universal bacteria primers, over 5,000 to 7,000-fold more bacteria were found in the skin of challenged non-vaccinated carp when compared with challenged vaccinated carp (**Figure 8A**). In gills, this difference decreased to 200 to 1,100-fold (**Supplementary Figure 9A**). The strong dysregulation of the bacterial flora in the skin compared to gills became particularly clear when individual bacterial groups were quantified. The abundance of flavobacteria was over 11,000-fold higher in the skin of challenged non-vaccinated carp compared to challenged vaccinated fish (**Figure 8A**), while in the gills of carp in this group, the abundance of these bacteria increased 72- to 670-fold (**Supplementary Figure 9A**). The same situation was seen in the case of aeromonads. The abundance of these bacteria increased in the skin of challenged non-vaccinated fish 14,000- to 62,000-fold (**Figure 8A**), while in gills, their abundance increased 28- to 188-fold when compared with challenged vaccinated carp (**Supplementary Figure 9A**). In the case of pseudomonas, the difference in abundance in the gills of vaccinated and non-vaccinated carp under challenge infection was 34- to 229-fold (**Supplementary Figure 9A**), while in skin, the difference in abundance was 1,400- to 3,900-fold (**Figure 8A**). The abundance of streptococci was more stable in both the skin and gills, and the differences were not larger than 150-fold (**Figure 8A** and **Supplementary Figure 9A**). When the 16S rRNA expression was quantified for all bacteria, for flavobacteria and for aeromonads, it was noted that the vaccination largely prevented an increase in bacteria rRNA levels in the skin and gills of carp under challenge infection with CyHV-3. In contrast to this, challenged non-vaccinated carp experienced extremely increased levels of bacteria rRNA in the skin and gills. Especially in carp treated with defensins as adjuvants, the expression of 16S rRNA for all bacteria increased in the skin 676,000-fold (**Figure 8B**), for flavobacteria 93,000-fold (**Figure 8C**) and for aeromonads 11,000,000-fold (**Figure 8D**). The differences between vaccinated and non-vaccinated and BSA-treated carp under challenge infection were much lower: from 6,000-fold in flavobacteria (**Figure 8C**), 8,600-fold in all bacteria (**Figure 8B**) to a 26,200-fold difference in the case of aeromonads (**Figure 8D**). In gills, the differences in bacterial rRNA levels were also much lower and ranged from 29-fold to 238-fold for the bacteria assays performed (**Supplementary Figures 9B–D**). The existence of secondary bacterial infections most likely exacerbated the pathology in the non-vaccinated CyHV-3 challenged fish and could contribute to the aberrant immune responses (e.g. elevated inflammation) in the skin and mortality.

## Discussion

Living in freshwater is a challenge for fish. All freshwater fish have body fluids with a higher osmolarity than the surrounding medium (11). The skin is rather impermeable to water and protects the fish from this osmotically-hostile environment, while the extensive area of the gill lamellae is optimised for gas exchange and allows ionic diffusion along the steep gradient from body fluids to the surrounding water (24). In addition, freshwater fish experience a constant influx of water, which is balanced by a renal excretion of substantial amounts of dilute urine. Carp under KHVD develop gill pathology, which manifests in a gradual destruction of the branchial epithelium. This includes hypertrophy, lifting and degeneration of epithelial cells (10, 25), but also a reduced expression of the genes encoding the secreted mucin (muc2like), the tight junction proteins, desmocollin 2, occludin and cytokeration 15 (present study). These changes indicate a significant impairment of the osmotic capacity of the gills and most likely enhance the osmotic efflux and impair the branchial uptake of ions, which would be required to compensate enhanced losses. Furthermore, large areas of damaged skin lacking the mucus cover can further reinforce the osmotic imbalance in KHVD-affected carp by increasing the passive influx of water (26). This could explain the substantial reduction in serum osmolarity and blood sodium concentration in KHVD-affected carp (10, 27), which most likely contribute to the pathology of this disease (10, 25). In the present study, skin and gill pathology as well as osmotic dysregulation developed in non-vaccinated carp under infection with a hyper-virulent CyHV-3 variant, but not in carp infected with the KHV-T ΔDUT/TK vaccine virus. While non-vaccinated carp under infection with CyHV-3 harboured a high virus load in the skin and gills, vaccinated carp were protected against high viremia, pathology and the severe ionic imbalance. Instead, in the gills of vaccinated carp, the expression of the gene encoding the tethered mucin 5b (*muc5b*) was enhanced, which could indicate a re-enforced mucus cover of the gills in these carp. In contrast, an increased ATPase1a activity (data not shown) was observed in the gills of non-vaccinated carp under infection with CyHV-3, which could represent a compensation for the passive loss of ions in CyHV-3 infected carp.

In the initial phase of infection, CyHV-3 is considered an epitheliotropic virus, with skin as the major portal of entry (28). Sensitive cells for virus infection and initial virus replication most likely are located in skin epidermis (29), with a subsequent internal spreading of the infection to various tissues of infected carp (20, 28). In an earlier study using a recombinant CyHV-3 strain with a double gene ORF56-57 deletion and luciferase-expressing cassette, virus infection and replication were mainly observed in the skin, while internal organs were virus positive at a low level over a short period of time (14). In the current study, the KHV-T ΔDUT/TK vaccine virus infected the skin of carp at a mean virus load of about 10^4^ DNA copies and subsequently spread to internal tissues with a mean virus load of about 10^3^ DNA copies. In contrast to this, in carp infected with the hyper-virulent wild type variant, the virus load increased to over 10^5^ copies in the skin and kidney, and this infection was accompanied with severe pathological changes in the skin, including detachment of mucus, loosening of scales and loss of the mucus and epidermis.

Fish are surrounded by a microbe-rich environment, which allows potentially pathogenic bacteria to disperse easily. In order to protect themselves from this plethora of facultative microbes, fish developed a mucosal infection barrier, which consists of a mucus layer and an intact skin epithelium (30). The mucus is vital for the protection of carp against CyHV-3 infection (29) and from further bacterial infections (31). CyHV-3 is known to damage the skin integrity by downregulating the expression of genes coding for mucins, antimicrobial peptides and cell junction proteins (9). The vaccination protected the carp from a rapid replication of the CyHV-3, and by this, from a deterioration of the skin epithelium. Therefore, our results show that vaccination against CyHV-3 can protect skin integrity of carp under infection with CyHV-3. Furthermore, we show that it prevents development of secondary bacterial infections (SBIs).

The importance of intact external surfaces for a protection against pathogens from surrounding water was shown in experiments with *rag1-*deficient zebrafish that lack adaptive immunity but have a similar survival rate to wild type fish as long as the mucosa is not disrupted (32, 33). In cases when the physical protective layer was stripped or damaged, the fish died within a period of a few weeks (34). In the current study, the epidermal layer was completely destroyed in vast areas of the skin in non-vaccinated carp under challenge with CyHV-3, and in other parts of the skin, the expression of genes encoding cell contact proteins and mucins was significantly downregulated. This deteriorated mucosal layer most likely failed to protect these carp from infection with opportunistic bacteria, which colonised the skin, gills and subsequently internal organs. The abundance of most bacterial species studied increased in the skin and to a lower extent in the gills of non-vaccinated carp during infection with CyHV-3 when compared to vaccinated carp. This may suggest that aeromonads, flavobacteria and pseudomonads are opportunistic facultative pathogens, which were waiting for suitable conditions to flourish. Fish mucus has a strong bactericidal effect (31) and its absence caused by CyHV-3 infection could create a biological niche allowing an uncontrolled growth of the bacteria. Protection against SBIs in vaccinated fish is particularly remarkable because during the challenge, the vaccinated fish swam in the same water with non-vaccinated carp which developed serious secondary infections, and were thus exposed to an increasing bacterial load and thus to an increasing infection pressure from potentially pathogenic bacteria. Nevertheless, CyHV-3-vaccinated fish did not develop bacterial infections. The likelihood of them developing SBI could be exacerbated by immunosuppression in the skin of non-vaccinated fish, which accompanied pronounced downregulation of marker genes for mucosa integrity. In particular, CD4 and CD8 T cell markers were downregulated, and both are important for antibacterial immunity (35). In parallel, KHVD in non-vaccinated carp was associated with uncontrolled inflammation and increased stress responses of the skin, indicated by an upregulation of the expression of *hsp70*. Evasion of immune responses by CyHV-3 has been documented multiple times (36–39). However, the immuno-modulating interplay between vaccination to primary viral pathogen and secondary bacterial pathogens has not been well studied in fish. The immersion of carp in the nanotube DNA vaccine against CyHV-3 showed induction of the non-specific immune enzyme activities, including lysozyme, superoxide dismutase, acidic and alkaline phosphatases, which could contribute to an antibacterial effect of the CyHV-3 vaccination (40).

The importance of maintaining a stable bacteria flora could be supported by the seemingly adverse effect of beta-defensins in the non-vaccinated challenged group, which can putatively be explained by disbalancing the bacteria flora during the vaccination process. The synthetic beta-defensins had both virucidal and bactericidal effects after refolding (data not shown), therefore the changes in the overall number of bacteria after vaccination in defensin groups could have been a hallmark of larger changes in bacteria composition, which later had an impact on the severity of SBI during the CyHV-3 challenge. On the other hand the lack of an adjuvant effect could be puzzling as well, because beta-defensins were shown to have a positive effect on the recruitment of antigen presenting cells and normally improve the efficacy of the vaccines (41–43). However, the tested vaccine already resulted in 100% relative percent survival (RPS) without adjuvant, therefore any beneficial effect of the adjuvant could have been masked by the high potency of the vaccine. As we tested only one application regime the use of synthetic beta-defensins as adjuvants has to be explored in future studies.

In order to better understand the interaction between antiviral vaccination and secondary bacterial infections, the current results could be interpolated in the light of influenza infections in humans and animal models. In humans, secondary bacterial infections are significant contributors to influenza-related mortality (44) and vaccination against the primary viral pathogen reduces the likelihood of lethal SBIs (45). Animal influenza models indicated that the development of secondary bacterial infection was facilitated by a disruption of epithelial surfaces. The influx of the bacteria exacerbated inflammation and induced a vicious cycle of elevated bacteria acquisition, followed by increased inflammatory responses (46). Vaccines targeted against viral proteins fostering mucosa disruption could prevent the development of these SBIs (47). Based on these observations, we could speculate that a similar situation is possible during KHVD where, yet unknown CyHV-3 virulence factors disrupt the skin mucosa of carp and a vaccination with a live-attenuated virus is so effective because a subsection of antibodies and other responses are targeted against these mucosa-disrupting virulence factors of the virus. Unfortunately, in fish, the impact of vaccination against viral infections on bacterial coinfection has been scarcely studied. Coinfections with parasitic sea lice were able to jeopardise the protective effect of a vaccination against *Piscirickettsia salmonis* in Atlantic salmon (48), although this research does not provide a comparable effect of a vaccination-related protection against secondary infections.

The existence of secondary infection raises a question of using supportive antibiotic treatments during infections with epitheliotropic viruses, which is another understudied subject in fish. Studies performed in mammals using less virulent viral pathogens like influenza showed that the antibiotic treatments can be used to limit the secondary bacteria induced mortality (49). In the case of CyHV-3 the antibiotic treatment is not scientifically tested and is not recognised as a mortality preventive measure. In koi the antibacterial treatments are suggested after fish survive a CyHV-3 infection but this is done on anecdotal basis and their outcome was not studied experimentally.

In vaccinated carp, 100% RPS was achieved. Vaccination not only prevented the mortality but also the development of the clinical signs of the disease. Vaccinated carp were largely protected against internal spreading of CyHV-3 from mucosal tissues to internal organs. This is in line with the findings of an earlier study using the same virus variant as vaccine, in which all vaccinated carp survived an infection with CyHV-3 as well and developed an increased level of antibodies binding to CyHV-3 in blood sera (15). Likewise, increased titres of virus neutralising antibodies were detected in sera from vaccinated and challenged carp in the present study. A comparable protective effect was also observed when carp were vaccinated with a Δ56-57 recombinant virus (14). While these live attenuated vaccine candidates provided 95%-100% RPS, different DNA single gene vaccines led to a typical survival rate of between 50-80%. This is a significant improvement compared to non-vaccinated carp, but in order to reach this high RPS, multiple immunisations were required (50–52). This suggests that currently live attenuated vaccines have an advantage over DNA vaccines.

The vaccination success using a recombinant attenuated virus variant was considered to rely on the induction of a protective mucosal immune response at the portal of entry (14). In the current study, vaccination with an attenuated virus indeed boosted B cell and T cell responses to some extent, as indicated by the upregulation of the typical marker genes coding for CD8, IgM and IgZ in skin. In addition, the expression of IgM and IgZ encoding genes was in particular induced in vaccinated carp under challenge infection. Therefore future studies should focus on testing the virus neutralisation capacity of the skin mucous. Besides this, the expression pattern of *mpo* was intriguing. The expression of this neutrophilic granulocytes marker was upregulated in vaccinated, non-challenged carp and the challenge infection with CyHV-3 induced an even higher response in the skin of the vaccinated carp. Recent results after vaccinating zebrafish against *Ichthyophthirius multifiliis* showed that neutrophils migrate to the infection site and remain there for a prolonged period of time even in the absence of the pathogen (53). In addition, the tuberculosis vaccine was shown to modulate and reprogram human neutrophil activity for at least 3 months post vaccination, providing some protection against other infections (54). Our results could indicate similar action in fish and this should also be addressed in further studies. Vaccination also increased the anti-inflammatory response (*il10*) and limited stress responses (*hsp70*) while providing a robust antiviral response (*irf7*, *mx2* and *trim21*), despite a much lower virus load. This suggests that vaccinating carp with the recombinant attenuated virus variant protects against aberrant immune responses, which increase the pathology. The uncontrolled inflammation induced in non-vaccinated carp under infection with the CyHV-3 could be accompanied by several disruptive factors like the generation of autoantibodies. These can then become the major part of the IgM generated by the host, resulting in “distraction” of immune responses from the real pathogens (55). These uncontrolled inflammatory responses in non-vaccinated carp under CyHV-3-infection could be related to the severe secondary bacterial infections observed in these carp.

## Conclusions

**Figure 9:**
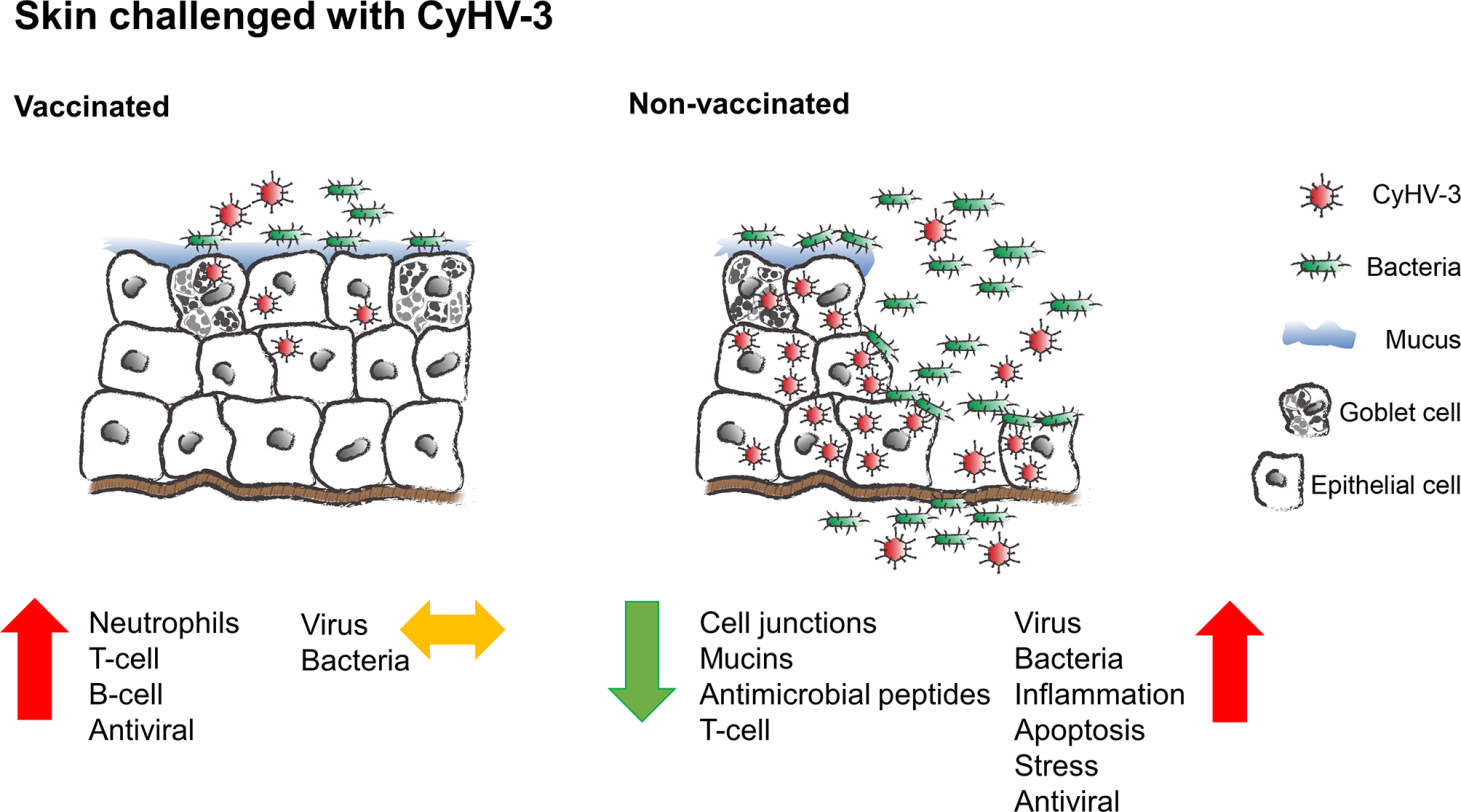
Schematic presentation of the protective effect of vaccination on skin of common carp upon CyHV-3 challenge.

By using a live-attenuated vaccine virus, we show that increased neutrophil, T and B cell responses provide protection of carp against CyHV-3 infection and lead to a preservation of gill and skin integrity, which supports a successful protection against osmotic disbalance and additional bacterial pathogens from the aquatic environment (**Figure 9**). CyHV-3-induced mortality in non-vaccinated carp was related to a strong osmotic disbalance, disruption of the mucosal barrier of the skin, and immunosuppression, leaving the way open for secondary bacterial pathogens. The impact of vaccination on protection against secondary infections should be studied in more detail, especially in the light of the fact that most diseases induced by epitheliotropic viruses might be accompanied by bacterial or parasitic coinfections.

## Data Availability Statement

The datasets presented in this study can be found in online repositories. The names of the repository/repositories and accession number(s) can be found in the article/**Supplementary Material**.

## Ethics Statement

The animal study was reviewed and approved by Local Ethical Committee in Lublin, Poland.

## Author Contributions

MiA, MM, and AR performed most of experiments and analyses with help from MS, AF, TK AF, MuA, JB, A-CM, FT, and VJ-S. MiA and AR performed additional analyses. LS and WF provided the vaccine, vaccination protocol and virus assays. MiA, MM, MR, and DS conceived the experimental design with contributions from AR. MiA and DS acquired the funding. MiA wrote the main body of the manuscript with contributions from all other authors. All authors corrected and approved the submitted version.

## Funding

Work was funded by German Federal Ministry of Food and Agriculture (BMEL) based on a decision of the Parliament of the Federal Republic of Germany, granted by the Federal Office for Agriculture and Food (BLE; grant number: 2815HS005). MiA was supported by the German Research Foundation (Deutsche Forschungsgemeinschaft, DFG) – project number 426513195. AF was supported by the Spanish Ministry of Economy and Competitiveness and European ERDF Funds (MCIU/AEI/FEDER, EU) (RTI2018-101969-J-I00). This publication was supported by Deutsche Forschungsgemeinschaft and University of Veterinary Medicine Hannover, Foundation within the funding programme Open Access Publishing.

## Conflict of Interest

The authors declare that the research was conducted in the absence of any commercial or financial relationships that could be construed as a potential conflict of interest.

## Publisher’s Note

All claims expressed in this article are solely those of the authors and do not necessarily represent those of their affiliated organizations, or those of the publisher, the editors and the reviewers. Any product that may be evaluated in this article, or claim that may be made by its manufacturer, is not guaranteed or endorsed by the publisher.
