# Supplementary material for: Don’t Let It Get Under Your Skin! – Vaccination Protects the Skin Barrier of Common Carp From Disruption Caused by Cyprinid Herpesvirus 3
Source: Front Immunol. 2022 Jan 31;13:787021. doi: 10.3389/fimmu.2022.787021 (PMC8842664; doi:10.3389/fimmu.2022.787021)
Supplement: Supplementary file 1 [file DataSheet_1.docx]

Supplementary Material

**Supplementary Table 1.** Primers used in the RT-qPCR.

| **Gene symbol** | **Gene product** | **Primer sequence 5’→3’ (sense, antisense)** | **NCBI accession code** |
| --- | --- | --- | --- |
| *rps11* | 40S ribosomal protein S11 | CCGTGGGTGACATCGTTACA TCAGGACATTGAACCTCACTGTCT | AB012087 |
| *ef1a* | Elongation factor 1-alpha | ACAACCCCAAGGCTCTCAA  CCGCCAACTTTCTTCTCAAC | AF485331 |
| *def1b* | Defensin beta 1 | CTTGCTTGTCCTTGTCGT  CCCTTGCCACAGCCTAA | JF343439 |
| *def3b* | Defensin beta 3 | CGGGTGAAGCTGATGACAC  TAAAATCGCAAAGCACAGCA | KF321773 |
| *cldn23* | Claudin 23 | AGGGAATCTGGGACATCTGC  GCTGGTGAGGATATGAGTGTACC | CA964745 |
| *cdh1* | Cadherin E | TCAGGACAGCTCCATTCAAG  AACAGCAGAGCCAAGATTCC | EC393368 |
| *ck15* | Cytokeratin 15 | CCTCCAAGTGTCCAAGACTG  GCAAGAGTTCCCTCAAGACC | EC393545 |
| *dsc2* | Desmocollin 2 | TGGCTTACTTGGGAGACGAG  GCCACAAGGCTACAACATCC | EC393171 |
| *muc2like* | Mucin 2-like | GCATCAACCTGCCATTCC  CAGCACAGTCGTCCACCAAG | MF380420 |
| *muc5b* | Mucin 5b | CAGCCCTCTTCCTCTTTCATC  CCACTCATCTTTCCTTTCTCTTC | JF343438 |
| *ocldn* | Occludin a | GGCTATGGAATGGGTGGAG  CGAGCAGAATGATGAAGGTG | EC393229 |
| *cd4* | CD4 molecule | CGTGGACATCTGGCTTTGTG  TTTGGTTTTGCGTCGTCTGT | DQ400124 |
| *cd8 b1* | CD8b1 molecule | CGGCTCGGAAACTATCACCT  GAGTGGCGGACAGGTTTTCTC | EU025120 |
| *igm* | Immunoglobulin heavy chain M | CACAAGGCGGGAAATGAAGA  GGAGGCACTATATCAACAGCA | AB004105 |
| *igz2* | Immunoglobulin heavy chain Z2 | AATTCTGAAGCACCTCACTAGA  CACACACATGAGAGACCCGAT | AB598368 |
| *inos* | Inducible nitric oxide synthase | CTGACTGGGTCTGGCTGGTG  CCTCCTCGCATTTCTCTTCTTG | AJ242906 |
| CyHV-3 ORF55 | CyHV-3 ORF55 | ACTTTATGCAGCAGCCCTTC CACTTCATGCACACCGCC  JOE-CCCATGGCGGACAAGCTGGACAAG -BHQ1 | AP008984 |
| CyHV-3 ORF89 | CyHV-3 ORF89 | GACGCCGGAGACCTTGTG  CGGGTTCTTATTTTTGTCCTTGTT  FAM-CTTCCTCTGCTCGGCGAGCACG-BHQ1 | AF411803 |
| *unbact16S* | All bacteria16S | AGGATTAGATACCCTGGAGTCCA  CATGCTCCACCGCTTGTGC | Multiple sequences |
| *aero16s* | Aeromonas 16S | GCGAAGGCGGCCCCCTGGACAAAGA  CCACGTCTCAAGGACACAGCCTCCAAATC | Multiple sequences |
| *flavo16s* | Flavobacterium 16S | GGGATAGCCCAGAGAAATTTGGAT  AGTCTTGGTAAGCCGTTACCTT | Multiple sequences |
| *pseud16s* | Pseudomonas 16S | TGCCTAGGAATCTGCCTGGTAGT  AATCCGACCTAGGCTCATCTGATAGCG | Multiple sequences |
| *strep16s* | Streptococcus 16S | CGGTAACTAACCAGAAAGGGA  ATAAATCCGGACAACGCTCGRAGA | Multiple sequences |

**Supplementary Table 2.** Results from the BestKeeper Software analysis of reference genes. * underlined bolded numbers indicate parameters with values over the acceptable limit.

| Sample set | Parameter | Gene |  |  |  |
| --- | --- | --- | --- | --- | --- |
| Skin Challenge integrated fluidic circuit (IFC) |  | *rps11* | *actb* | *gapdh* | *ef1a* |
|  | geom. Mean [Ct] | 9,878 | 10,275 | 11,603 | 8,431 |
|  | ar. Mean [Ct] | 9,944 | 10,354 | 11,762 | 8,516 |
|  | min [Ct] | 8,700 | 8,021 | 7,923 | 7,041 |
|  | max [Ct] | 15,160 | 13,908 | 16,663 | 14,721 |
|  | std dev [± Ct] | 0,874 | 0,967 | **1,611*** | 0,925 |
|  | CV [% Ct] | 8,786 | 9,339 | 13,694 | 10,862 |
|  |  |  |  |  |  |
| Skin Vaccination RT-qPCR |  | *rps11* | *actb* | *gapdh* | *ef1a* |
|  | geom. Mean [Ct] | 17,968 | - | - | 18,955 |
|  | ar. Mean [Ct] | 17,997 | - | - | 18,989 |
|  | min [Ct] | 13,707 | - | - | 13,981 |
|  | max [Ct] | 20,495 | - | - | 22,051 |
|  | std dev [± Ct] | 0,732 | - | - | 0,781 |
|  | CV [% Ct] | 4,067 | - | - | 4,115 |
|  |  |  |  |  |  |
| Kidney Vaccination RT-qPCR |  | *rps11* | *actb* | *gapdh* | *ef1a* |
|  | geom. Mean [Ct] | 19,852 | - | - | 21,109 |
|  | ar. Mean [Ct] | 19,862 | - | - | 21,178 |
|  | min [Ct] | 17,537 | - | - | 10,080 |
|  | max [Ct] | 21,359 | - | - | 23,136 |
|  | std dev [± Ct] | 0,410 | - | - | 0,669 |
|  | CV [% Ct] | 2,067 | - | - | 3,159 |
|  |  |  |  |  |  |
| Skin Challenge RT-qPCR |  | *rps11* | *actb* | *gapdh* | *ef1a* |
|  | geom. Mean [Ct] | 17,433 | - | - | 18,896 |
|  | ar. Mean [Ct] | 17,465 | - | - | 18,946 |
|  | min [Ct] | 13,046 | - | - | 12,772 |
|  | max [Ct] | 19,917 | - | - | 21,707 |
|  | std dev [± Ct] | 0,794 | - | - | 0,997 |
|  | CV [% Ct] | 4,544 | - | - | 5,262 |
|  |  |  |  |  |  |
| Kidney Challenge RT-qPCR |  | *rps11* | *actb* | *gapdh* | *ef1a* |
|  | geom. Mean [Ct] | 19,517 | - | - | 20,658 |
|  | ar. Mean [Ct] | 19,537 | - | - | 20,692 |
|  | min [Ct] | 16,471 | - | - | 16,706 |
|  | max [Ct] | 21,421 | - | - | 23,162 |
|  | std dev [± Ct] | 0,652 | - | - | 0,919 |
|  | CV [% Ct] | 3,335 | - | - | 4,442 |
|  |  |  |  |  |  |
| Gill Challenge RT-qPCR |  | *rps11* | *actb* | *gapdh* | *ef1a* |
|  | geom. Mean [Ct] | 17,854 | - | - | 19,471 |
|  | ar. Mean [Ct] | 17,857 | - | - | 19,478 |
|  | min [Ct] | 16,940 | - | - | 18,004 |
|  | max [Ct] | 18,758 | - | - | 20,714 |
|  | std dev [± Ct] | 0,258 | - | - | 0,392 |
|  | CV [% Ct] | 1,445 | - | - | 2,013 |

**Supplementary Table 3.** Primers used in the integrated fluidic circuit (IFC) nanoscale RT-qPCR.

| **Gene symbol** | **Gene product** | **Primer sequence 5’→3’ (sense, antisense)** | **NCBI accession code** | **Fragment length [bp]** | **Primer efficieny [%]** |
| --- | --- | --- | --- | --- | --- |
| *actb* | Cytoplasmic actin 1 | GCATCAGGGAGTGATGGTT  GGCCTCATCTCCCACATAG | XM_019089432 | 58 | (Reference gene) |
| *afap1* | Apoptotic protease activating factor 1 | CCTCTGTCCAGGCGATTCTGA  AGCCAGCCATGCCAAATACAGT | EU490407 | 150 | 97.4 |
| *bf/c2* | Bf/c2 complement component | CGTATCGGAGACAAAGTGC  GTGTACTGGGCATAGCAGC | AB047361 | 125 | 93.4 |
| *c1rs* | Complement C1s-A subcomponent | CAGATCCAGCTAACGTTCAATC  ACACAACCGAGACCGAATC | AB042609 | 76 | 100.2 |
| *c3* | Complement component C3 | AGCATATGTGGCCAAAGTCTTCA  AATCCTCTTTAAAGGAGCCGTATA | AB016215 | 128 | 95.9 |
| *casp6* | Caspase 6 | ATGCCGTAGCCTTGTAGGG  ACGACAAGCCTGCAAGAT | XM_019079508 | 52 | 97.8 |
| *casp9* | Caspase 9 | TTCTGAAACTAAAGGGGTGAGA  CTGGCATCCATCTTATAACACTG | EC394517, XM_019117197 | 63 | 88.3 |
| *cd4* | CD4 molecule | ACCGAGAGTGAAGGTAGAAGAAA  TTCACGTCTTTCGCCCTTTGGA | DQ400124 | 123 | 100.6 |
| *cd8a1*  (*LOC109054692*) | CD8a1 molecule | CCAAAAATTGCTCCATTACC  CTGGGTTTGCCTTGTTTC | EU025118 | 83 | 101.7 |
| *cd8a2* | CD8a2 molecule | CAGCCATGAACAACAATAAACTC  TTTTTGTGAAATCTATACTGGTTCTC | EU025119 | 77 | 100.5 |
| *cd8b1*  (*LOC109045157*) | CD8b1 molecule | GGCTTCTATTCCTGCATGTTTA  AGGATTCACTCCAGGCATTAT | EU025120 | 75 | 86.7 |
| *cd8b2* | CD8b2 molecule | GGCTTCTATTCCTGCATGTTTA  AGGATTCACTCCAGGCATTAT | EU025121 | 75 | 96.0 |
| *ch25h_b* (*LOC109099495*) | Cholesterol 25-hydroxylase-like protein b | CCTGCCTGCTTCTCTTTGA  ACCTTATGGAAAGTGCGGTAC | JZ503992, XM_019113012, XM_019113007 | 96 | 96.8 |
| *ch25h_a* (*LOC109082232*) | Cholesterol 25-hydroxylase-like protein a | ACCACCTGGGCATTCATG  CGCCCAAGGGAAGTCATA | XM_019097130 | 75 | 89.2 |
| *crp1* | C-reactive protein 1 | TGAACAGACCTTCCCTCAGA  TCTTGCTGCAGGTAAAGCTC | JQ010977 | 51 | 93.0 |
| *crp2* | C-reactive protein 2 | TGAAGTGGGCCTCAGTGGTAAA  CAACACGCATGCAGAGAGTAAAT | JQ010978 | 59 | 97.6 |
| *cyp7b1* | Cytochrome P450 7B1 | CAGCAGTTTCTTTCACAACTACA  TGAATGTAATGTACCTTCCTGCTAT | XM_019086682, XM_019086681 | 79 | 101.8 |
| *eef1a1* | Elongation factor 1-alpha 1 | ATTGGAGGTATTGGAACCGT  CAAAGGTCACAACCATACCTG | AF485331 | 76 | (Reference gene) |
| *fdps* | Farnesyl pyrophosphate synthase | GAGGGCAGAACTAGAGGCAT  CCGTTCAACACTTTCAGCA | CA964878, XM_019091227 | 55 | 115.6 |
| *fel* | Fish-egg lectin | GCGGAGCAATGAAGGTTT  GTACAGTCTAAAGCCAGAGTGTGA | XM_019114883 | 79 | 98.6 |
| *gapdh* | Glyceraldehyde-3-phosphate dehydrogenase | GCAAGCTGGTCATTGACG  GTTGGCTGGATCCCTCTC | AJ870982 | 59 | (Reference gene) |
| *gig1* | Interferon-inducible protein | TCAGAATCTCCCATACTATGAGG  AATAATGCGATCCTTGTTGCTGC | XM_019073246, XM_019111114 | 121 | 100.3 |
| *gpr183* | Cyprinus carpio G protein-coupled receptor 183a | GGTGTGTTAGAAAACGTGGTTAAT  ATGGGGTCAGTTATGAAATCAA | XM_019109670 | 133 | 98.1 |
| *hsp70 (hsp1a1)* | Heat-shock 70 kDa protein-like protein | CGAGGCCATCAGCTGGCTAG  TGCTCCACGTGCCTGAGCTC | JN544930 | 157 | 101.4 |
| *iap* | Baculoviral IAP repeat-containing protein 2-like protein | TTGGGAGTCTGGTGATGATC  CTTGCAACAGGTATTCACATCTT | XM_019075075, XM_019075070, XM_019075064 | 71 | 101.5 |
| *ifna3-1 (LOC109059985)* | Interferon alpha-3-like | TTAGATCAGATCATCAGCCTCAT  TTTGGTATTGGGCCACAC | AB376667, AB376667 | 139 | 98.3 |
| *igna3-2 (LOC109046410)* | Interferon alpha-3-like | ATGGATCAGGTTACTGGTGTAGA  CCTCGAAAATCTTTCCACTTT | XM_019064164 | 128 | 100.6 |
| *igm* | Immunoglobulin heavy chain | CGAATATGCAGTTCCTATTCAAGAT  GGGGCATCAAGTTCCACATGTT | AB004105 | 216 | 97.6 |
| *igz-1* | Immunoglobulin heavy chain Z1 | GGTTGTGACTCTGTATTCGTAGA  CAATTATTGTGTACAGAGCCAGTT | AB598367 | 136 | 92.6 |
| *igz-2* | Immunoglobulin heavy chain Z2 | GAAGGATACAAATGCTCCGTTAC  ATAACAAGTGAGCAACGGGACAA | AB598368 | 133 | 98.6 |
| *il10* | Interleukin 10 | ACTTGGAACCATTATTGAATGAA  GACGTTACATCCATAAGGACTATTG | AB110780 | 65 | 99.7 |
| *il12p35* | Interleukin-12 p35 subunit | AACCCAGAGCTCACCAATAGT  AGCAGTTCTGCAGGAGGTC | AJ580354 | 58 | 86.6 |
| *il1b1* | Interleukin-1 beta | CCCAGATCAACTAGGCATGATG  TTCAACTTTTCCACAGCGATGAC | AJ245635 | 123 | 94.3 |
| *inos2b* | Inducible nitric oxide synthase 2b | AGAAAGTTGCACTGCTGAAAA  GGTTCCCCATATTTACCTTTGT | AJ242906 | 61 | 99.9 |
| *irf3* | Interferon regulatory factor 3-like protein | CCGCGAATCCACATAAAGT  AGATCCCAAAGCTGCAGAA | JQ478481 | 62 | 103.3 |
| *irf7* | Interferon regulatory factor 7-like protein | TCAAAACCACCAAGAGATCC  CAGACATGTAATTAGCTGCAGTG | JQ698666 | 86 | 90.6 |
| *masp2* | Mannan-binding lectin serine peptidase 2 | GGACCAGAGGGACACAGATT  CCTTCTGAAAACACCTGTACG | AB234294 | 98 | 93.8 |
| *mpo* | Myeloid-specific peroxidase | TACCAGGCTATAATGCATGGCG  GCGATGCCTCCCAACCAAATG | AB429306 | 157 | 100.0 |
| *mx2* | Interferon-induced GTP-binding protein MxB-like | AGGGAAGAGTTCTGTTCTCGA  GGCATCTTGTAACAATTCCACTA | XM_019081222 | 74 | 97.7 |
| *orf92* | Orf92 (CyHV-3) | GCGAGGTAGGTTTCGGTATCAA  CCCGCTGCGCCACCTTATAAA | DQ177346 | 95 | (Virus detection) |
| *p53* | Tumor protein p53 | GTCGATGTCCCCATCATG  AGGAGGTGCCAATCCATC | XM_019097618, XM_019097614, XM_019097608, XM_019097598 | 53 | 99.9 |
| *pkz* | Calcium/calmodulin-dependent protein kinase kinase 2 | ATCAGTTCCTGGCTAAGTGTGC  ATCTCCTCCAGCGTGCTAACAA | XM_019063422 | 172 | 96.5 |
| *rps11* | 40S ribosomal protein S11 | AAGGAGAAGCTCCCACGTTA  TGCCAGCAATAGCCTCTCT | AB012087 | 73 | (Reference gene) |
| *serpinb1l3* | Serpin peptidase inhibitor, clade B (ovalbumin), member 1, like 3 | GGTGTGTCTGCTGCTCATATAA  CTGACACAATGCTGTAAAACG | DC996878 | 96 | 98.0 |
| *tcra-1* | T cell antigen receptor alpha chain C region 1 | AACCACCAGCCTTTTACAAATTCA  AGTTTTCTGATCCACTAAAGCTGA | EU025122 | 153 | 96.2 |
| *tcra-2* | T cell antigen receptor alpha chain C region 2 | TAAAAAAGAAGAACCGCCTGTCTA  CTGCTGTAATAACTTCGTTCTGC | EU025123 | 132 | 100.8 |
| *trim21* | E3 ubiquitin-protein ligase TRIM21-like | CTTTGTGCAGTAAACGTGACTCT  TCTTCCCGAAGTGATGTGAGGT | XM_019085545 | 174 | 98.7 |
| *vig1* | Viperin | CGGGACTATAAGGTGGCTTT  AAGACCTTCCAGCGCACT | JX131617, EX881775, EX880905 | 110 | 89.6 |


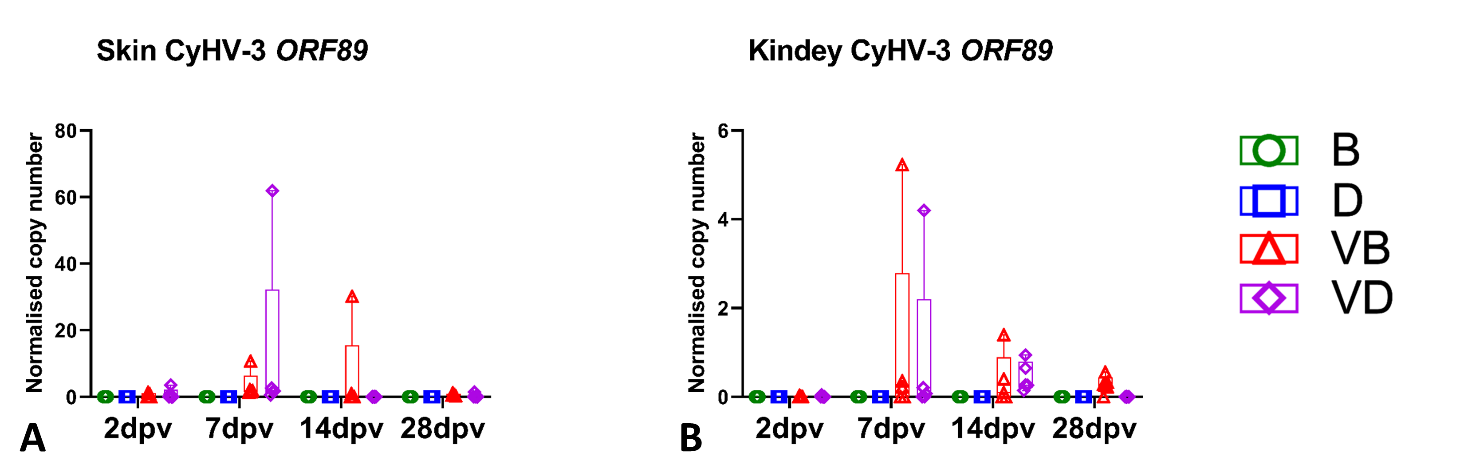


**Supplementary Figure 1.** Levels of transcripts of CyHV-3 *ORF89* after vaccination, measured with RT-qPCR in A) skin: B) kidney. The description of experimental groups: B – BSA non-vaccinated, D – defensins non-vaccinated, VB - BSA vaccinated, VD – defensins vaccinated. The results are presented as 25%-75% box plots with min. and max. values as whiskers with indication of all data points. The data are shown as normalised copy numbers. * indicates statistical significant difference at *p* < 0.05. Statistical analysis was performed with two-way ANOVA with multiple comparisons test performed with the Holm-Sidak method.


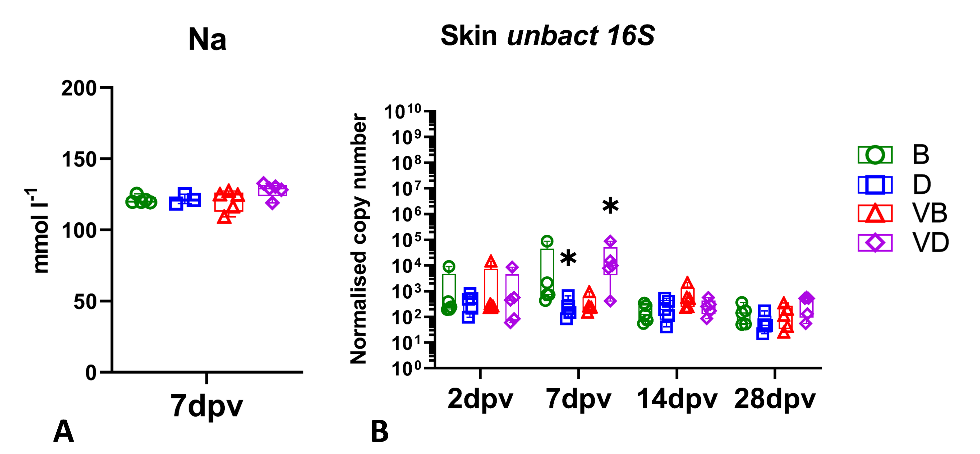


**Supplementary Figure 2.** Sodium level in blood serum after vaccination at 7 dpv (A) and level of bacterial 16S rRNA for all bacteria after vaccination, measured with RT-qPCR (B). The description of experimental groups: B – BSA non-vaccinated, D – defensins non-vaccinated, VB - BSA vaccinated, VD – defensins vaccinated. The results are presented as 25%-75% box plots with min. and max. values as whiskers with indication of all data points. The 16S data are shown as normalised copy. * indicates statistical significant difference at *p* < 0.05. Statistical analysis was performed with one-way or two-way ANOVA with multiple comparisons test performed with the Holm-Sidak method.

**
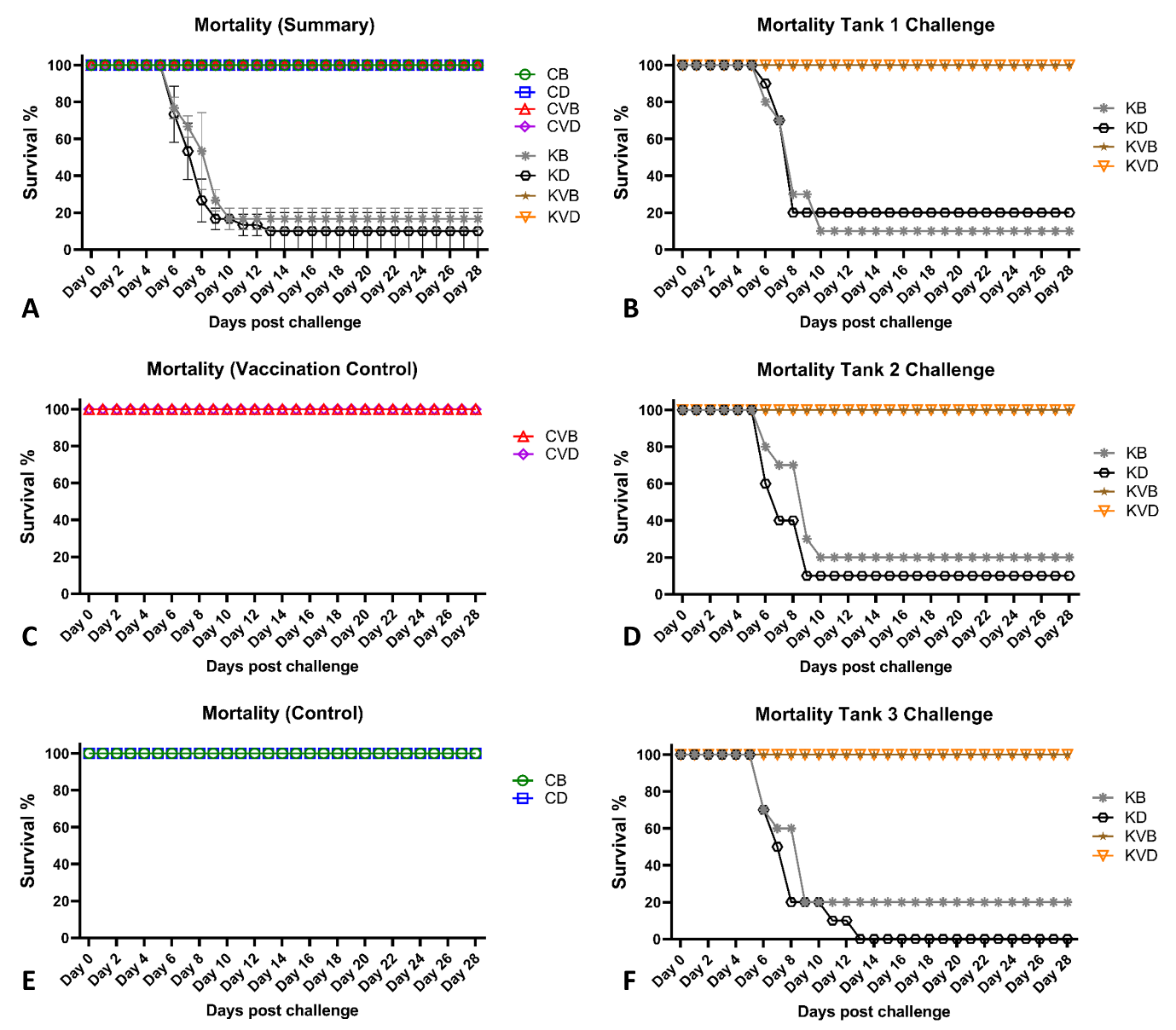
**

**Supplementary Figure 3.** Mortality curves during the challenge with CyHV-3. A) Summary of approximated mortality during challenge. B) Approximated mortality in Tank 1 after challenge with CyHV-3. C) Approximated mortality in tank with mock challenged vaccinated fish, D) Approximated mortality in Tank 2 after challenge with CyHV-3. E) Approximated mortality in tank with mock challenged non-vaccinated fish. F) Approximated mortality in Tank 3 after challenge with CyHV-3. The description of experimental groups: CB – BSA, non-vaccinated, unchallenged control, CD – defensins, non-vaccinated, unchallenged control, CVB – BSA, vaccinated, unchallenged control, CVD – defensins, vaccinated, unchallenged control, KB - BSA non-vaccinated, CyHV-3 challenged, KD – defensins non-vaccinated, CyHV-3 challenged, KVB – BSA, vaccinated, CyHV-3 challenged, KVD – defensins, vaccinated, CyHV-3 challenged. The results are presented as lines indicating the presence of live fish in the tank.


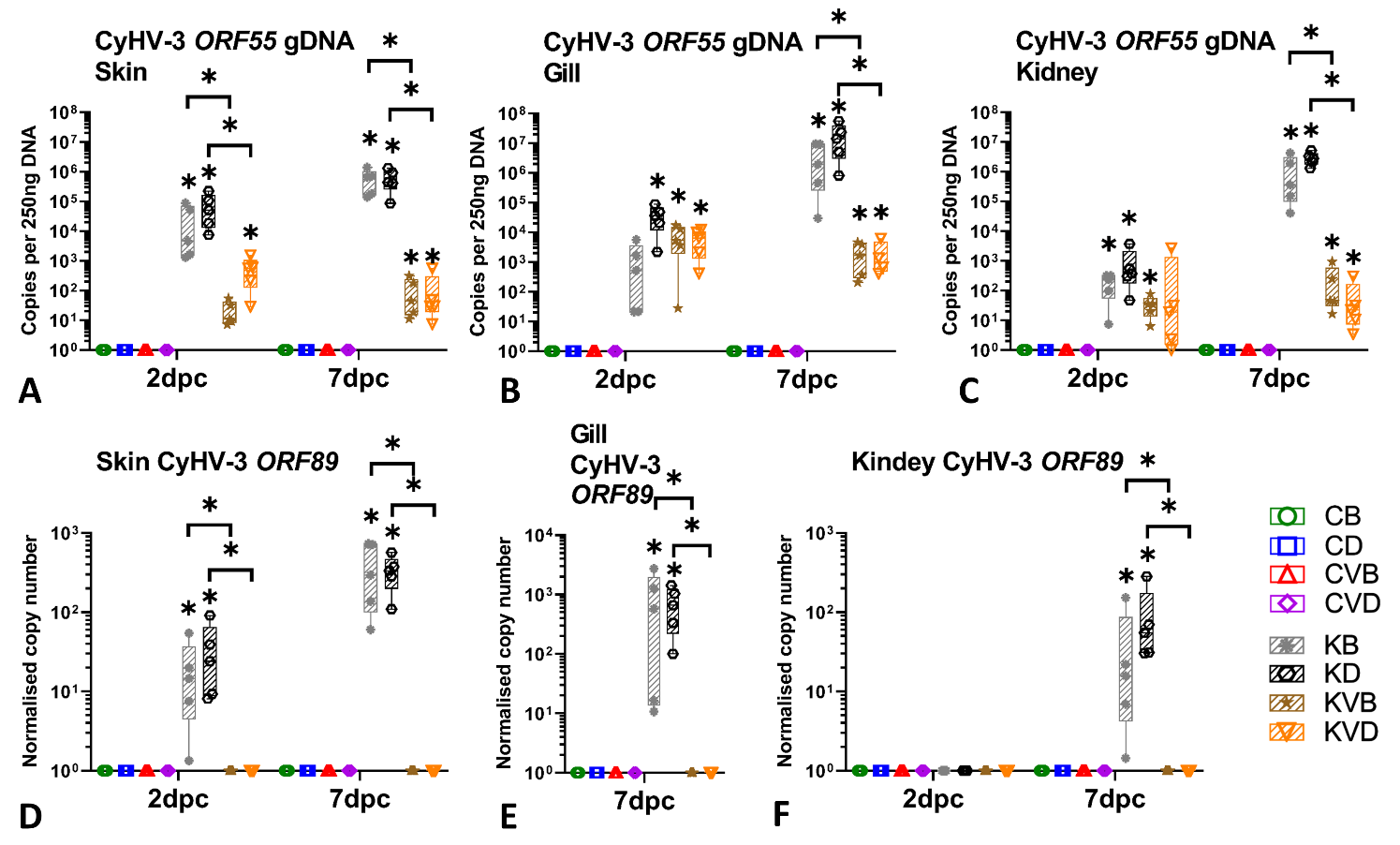


**Supplementary Figure 4.** Virus load based on CyHV-3 ORF55 in A) skin, B) gills, C) kidney at 2 dpc and 7 dpc. Level of transcripts of CyHV-3 *ORF89* after CyHV-3 challenge measured with RT-qPCR in A) skin, B) gills, C) kidney. The description of experimental groups: CB – BSA, non-vaccinated, unchallenged control, CD – defensins, non-vaccinated, unchallenged control, CVB – BSA, vaccinated, unchallenged control, CVD – defensins, vaccinated, unchallenged control, KB - BSA non-vaccinated, CyHV-3 challenged, KD – defensins non-vaccinated, CyHV-3 challenged, KVB – BSA, vaccinated, CyHV-3 challenged, KVD – defensins, vaccinated, CyHV-3 challenged. The results are presented as 25%-75% box plots with min. and max. values as whiskers with indication of all data points. The data are shown as normalised copy numbers. * indicates statistical significant difference at *p* < 0.05. Statistical analysis was performed with two-way ANOVA with multiple comparisons test performed with the Holm-Sidak method.

**
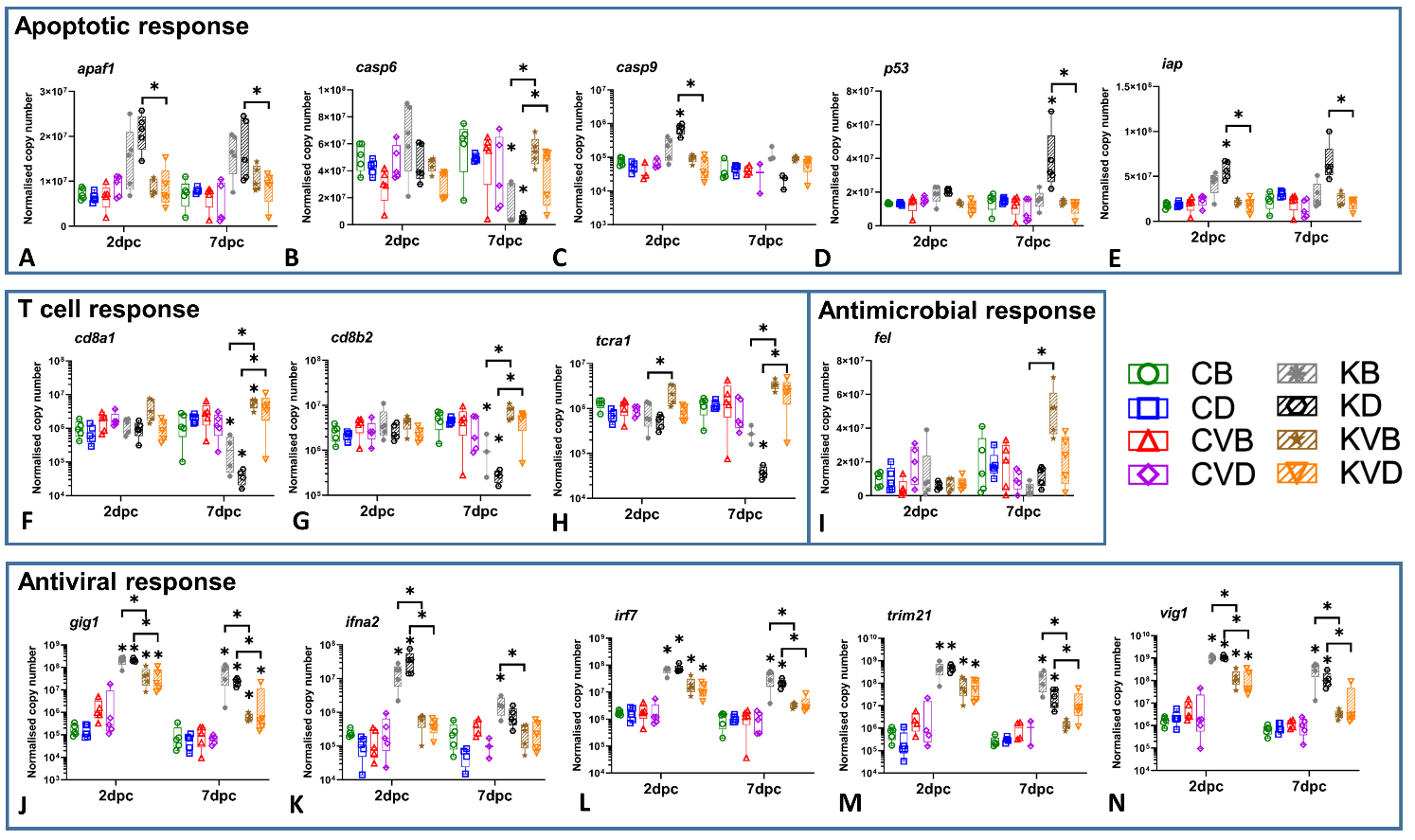
**

**Supplementary Figure 5.** Level of transcripts coding for proteins involved in immune responses after the CyHV-3 challenge with significantly regulated response at 2 dpc and 7 dpc measured with integrated fluidic circuit nanoscale RT-qPCR in skin. Presented are markers for apoptotic response: A) *apaf1*, B) *casp6*, C) *casp9*, D) *p53*, E) *iap*, T cell response F) *cd8a1*, G) *cd8b2*, H) *tcra1,* antimicrobial response: I) *fel*, antiviral response: J) *gig1*, K) *ifna2*, L) *irf7*, M) *trim21*, N) *vig1*. The description of experimental groups: CB – BSA, non-vaccinated, unchallenged control, CD – defensins, non-vaccinated, unchallenged control, CVB – BSA, vaccinated, unchallenged control, CVD – defensins, vaccinated, unchallenged control, KB - BSA non-vaccinated, CyHV-3 challenged, KD – defensins non-vaccinated, CyHV-3 challenged, KVB – BSA, vaccinated, CyHV-3 challenged, KVD – defensins, vaccinated, CyHV-3 challenged. The results are presented as 25%-75% box plots with min. and max. values as whiskers with indication of all data points. The data are shown as normalised copy numbers. * indicates statistical significant difference at *p* < 0.05. Statistical analysis was performed with two-way ANOVA with multiple comparisons test performed with the Holm-Sidak method.


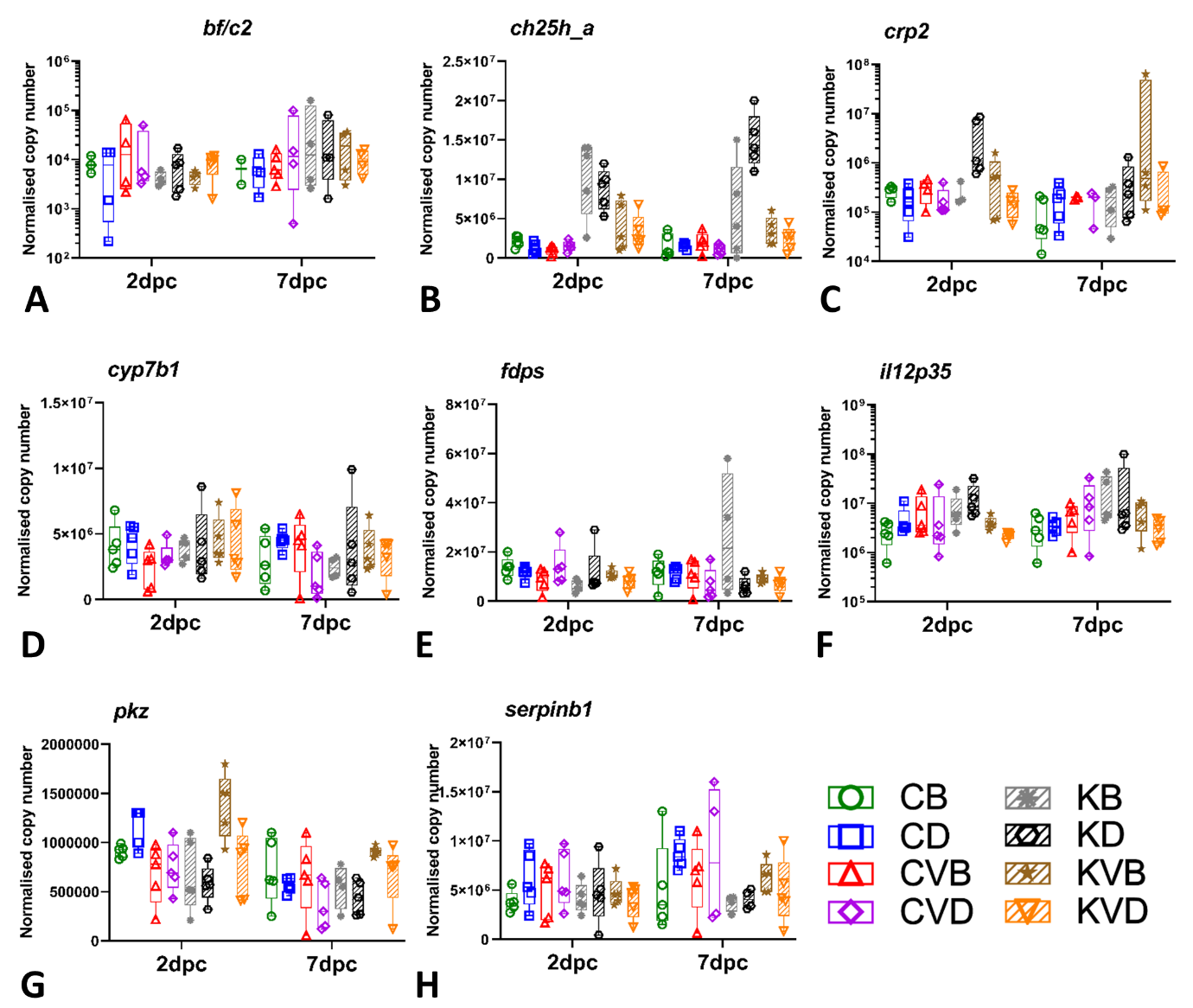


**Supplementary Figure 6.** Level of transcripts coding for proteins involved in immune responses after the CyHV-3 challenge with non-significantly regulated response at 2 dpc and 7 dpc measured with integrated fluidic circuit nanoscale RT-qPCR in skin: A) *bf/c2*, B) *ch25h_a*, C) *crp2*, D) *cyp7b1*, E) *fdps*, F) *il12p35*, G) *pkz*, H) *serpinb1*. The description of experimental groups: CB – BSA, non-vaccinated, unchallenged control, CD – defensins, non-vaccinated, unchallenged control, CVB – BSA, vaccinated, unchallenged control, CVD – defensins, vaccinated, unchallenged control, KB - BSA non-vaccinated, CyHV-3 challenged, KD – defensins non-vaccinated, CyHV-3 challenged, KVB – BSA, vaccinated, CyHV-3 challenged, KVD – defensins, vaccinated, CyHV-3 challenged. The results are presented as 25%-75% box plots with min. and max. values as whiskers with indication of all data points. The data are shown as normalised copy numbers. Statistical analysis was performed with two-way ANOVA with multiple comparisons test performed with the Holm-Sidak method.


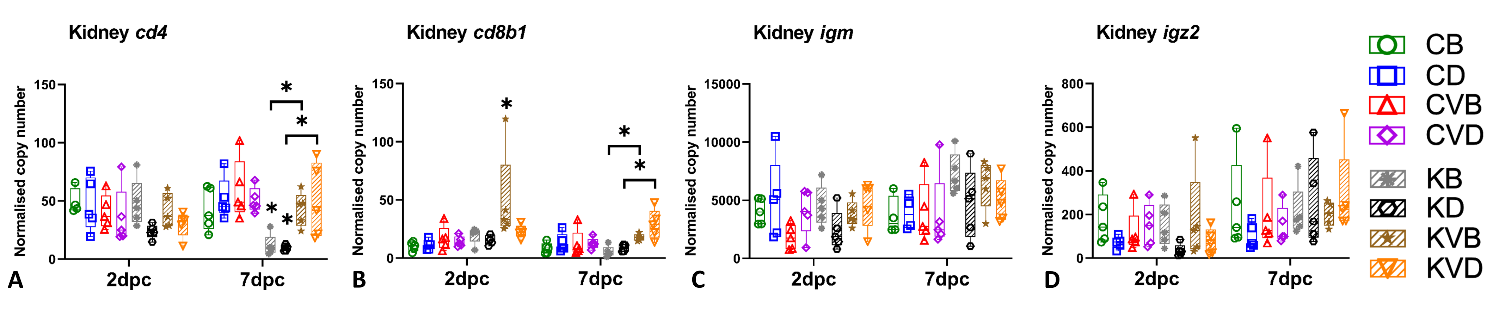


**Supplementary Figure 7.** Level of transcripts coding for proteins involved in immune responses measured with RT-qPCR after CyHV-3 challenge in kidney: A) *cd4*, B) *cd8b1*, C) *igm*, D) *igz2*. The description of experimental groups: CB – BSA, non-vaccinated, unchallenged control, CD – defensins, non-vaccinated, unchallenged control, CVB – BSA, vaccinated, unchallenged control, CVD – defensins, vaccinated, unchallenged control, KB - BSA non-vaccinated, CyHV-3 challenged, KD – defensins non-vaccinated, CyHV-3 challenged, KVB – BSA, vaccinated, CyHV-3 challenged, KVD – defensins, vaccinated, CyHV-3 challenged. The results are presented as 25%-75% box plots with min. and max. values as whiskers with indication of all data points. The data are shown as normalised copy numbers. * indicates statistical significant difference at *p* < 0.05. Statistical analysis was performed with two-way ANOVA with multiple comparisons test performed with the Holm-Sidak method.


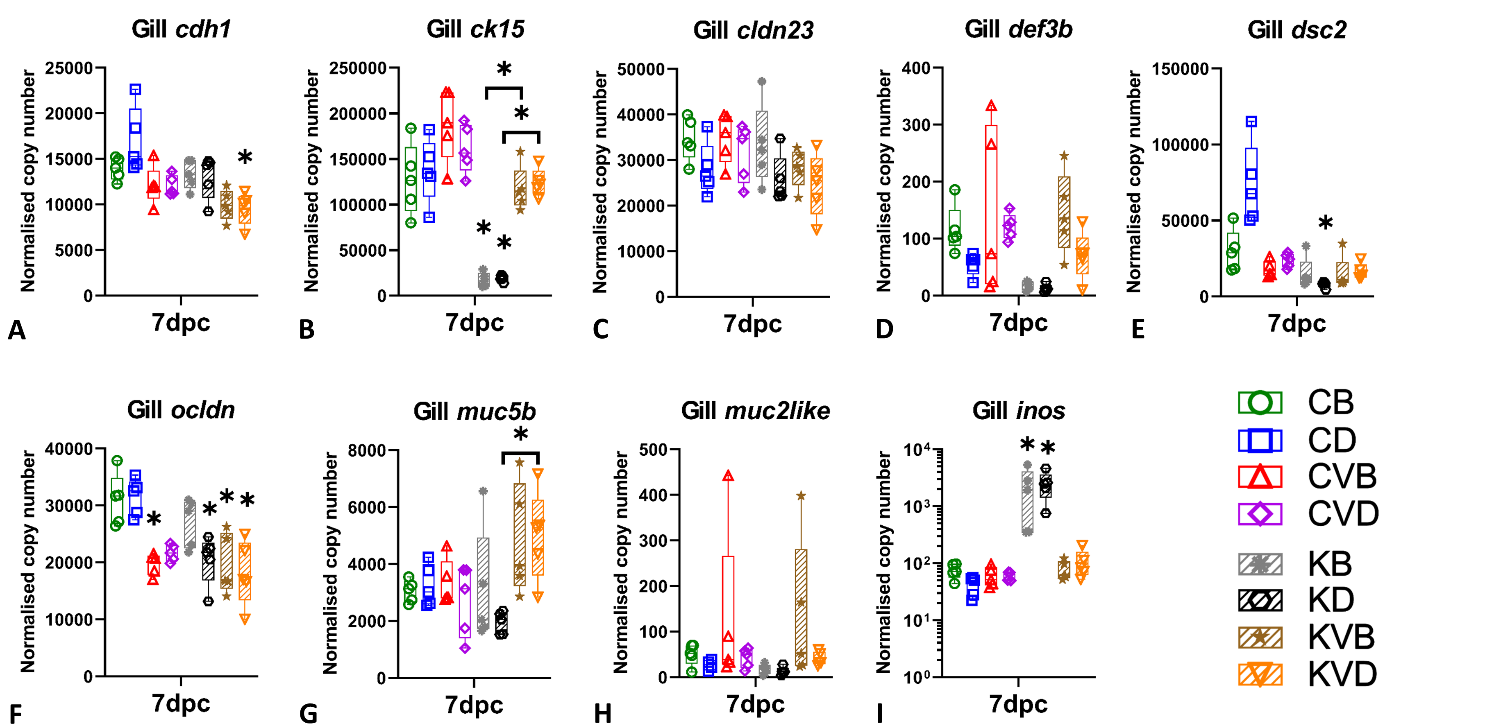


**Supplementary Figure 8.** Level of transcripts coding for proteins involved in gills barrier integrity and function after the CyHV-3 challenge at 7 dpc measured with RT-qPCR: A) *cdh1*, B) *ck15*, C) *cldn23*, D) *dsc2*, E) *def3b*, F) *muc2like*, G) *muc5b*, H) *ocldn* I) *inos*. The description of experimental groups: CB – BSA, non-vaccinated, unchallenged control, CD – defensins, non-vaccinated, unchallenged control, CVB – BSA, vaccinated, unchallenged control, CVD – defensins, vaccinated, unchallenged control, KB - BSA non-vaccinated, CyHV-3 challenged, KD – defensins non-vaccinated, CyHV-3 challenged, KVB – BSA, vaccinated, CyHV-3 challenged, KVD – defensins, vaccinated, CyHV-3 challenged. The results are presented as 25%-75% box plots with min. and max. values as whiskers with indication of all data points. The data are shown as normalised copy numbers calculated. * indicates statistical significant difference at *p* < 0.05. Statistical analysis was performed with two-way ANOVA with multiple comparisons test performed with the Holm-Sidak method.


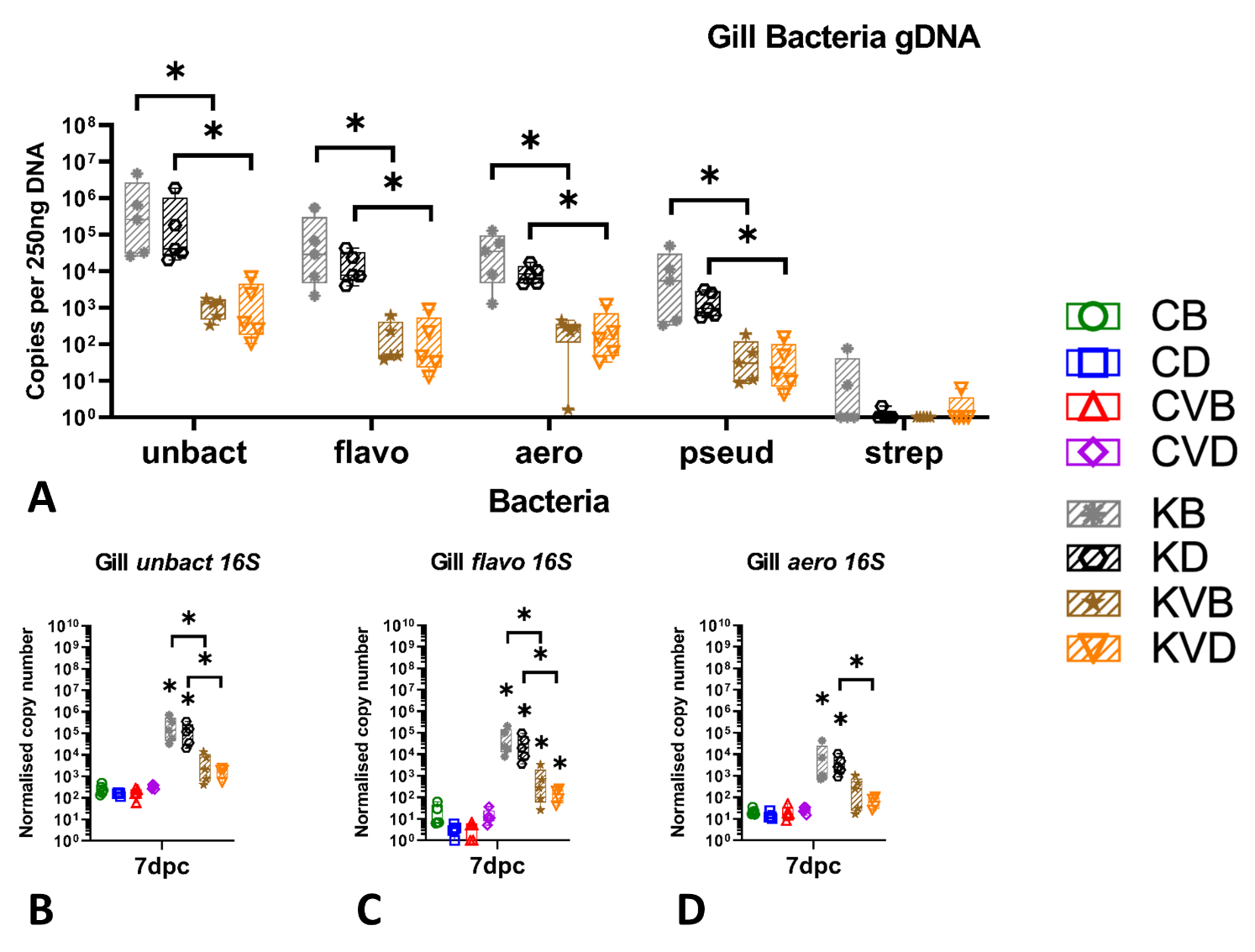


**Supplementary Figure 9.** Bacterial load and level 16S rRNA for selected bacteria species after challenge with CyHV-3 measured with qPCR or RT-qPCR. A) All bacteria (unbact), flavobacteria (flavo), aeromonads (aero), pseudomonads (pseud) and streptococci (strep) load in gill at 7 dpc. Expression of bacterial 16S rRNA: B) all bacteria 16S in gills at 7 dpc, C) floavobacteria 16S in gills at 7 dpc, D) aeromonads 16S in gills at 7 dpc. The description of experimental groups: CB – BSA, non-vaccinated, unchallenged control, CD – defensins, non-vaccinated, unchallenged control, CVB – BSA, vaccinated, unchallenged control, CVD – defensins, vaccinated, unchallenged control, KB - BSA non-vaccinated, CyHV-3 challenged, KD – defensins non-vaccinated, CyHV-3 challenged, KVB – BSA, vaccinated, CyHV-3 challenged, KVD – defensins, vaccinated, CyHV-3 challenged. The results are presented as 25%-75% box plots with min. and max. values as whiskers with indication of all data points. Bacteria load data are shown as genomic 16S copy numbers normalised for 250 ng of extracted DNA. The expression data are shown as normalised copy numbers. * indicates statistical significant difference at *p* < 0.05. Statistical analysis was performed with two-way ANOVA with multiple comparisons test performed with the Holm-Sidak method.
